# ^68^Ga-MY6349 PET/CT imaging to assess Trop2 expression in multiple types of cancer

**DOI:** 10.1172/JCI185408

**Published:** 2024-11-07

**Authors:** Haojun Chen, Liang Zhao, Yizhen Pang, Jiyun Shi, Hannan Gao, Yining Sun, Jianhao Chen, Hao Fu, Jiayu Cai, Lingyu Yu, Ru Zeng, Long Sun, Hua Wu, Zhanxiang Wang, Fan Wang

**Affiliations:** 1 Department of Nuclear Medicine and Minnan PET Center, Xiamen Key Laboratory of Radiopharmaceuticals, The First Affiliated Hospital of Xiamen University, School of Medicine, Xiamen University, Xiamen, China.; 2 Xiamen Key Laboratory of Rare Earth Photoelectric Functional Materials, Xiamen Institute of Rare Earth Materials, Haixi Institute, Chinese Academy of Sciences, Xiamen, China.; 3 Departments of Diagnostic Radiology, Surgery, Chemical and Biomolecular Engineering, and Biomedical Engineering, Yong Loo Lin School of Medicine and College of Design and Engineering, National University of Singapore, Singapore.; 4 Clinical Imaging Research Centre, Centre for Translational Medicine, Yong Loo Lin School of Medicine, National University of Singapore, Singapore.; 5 Key Laboratory of Biomacromolecules, CAS Center for Excellence in Biomacromolecules, Institute of Biophysics, Chinese Academy of Sciences, Beijing, China.; 6 Department of Medical Oncology, The First Affiliated Hospital of Xiamen University, Xiamen, China.; 7 Department of Neurosurgery and Department of Neuroscience, Fujian Key Laboratory of Brain Tumors Diagnosis and Precision Treatment, Xiamen Key Laboratory of Brain Center, The First Affiliated Hospital of Xiamen University, School of Medicine, Xiamen University, Xiamen, China.; 8 Medical Isotopes Research Center and Department of Radiation Medicine, School of Basic Medical Sciences, International Cancer Institute, Peking University, Beijing, China.; 9 Guangzhou National Laboratory, Guangzhou, China.

**Keywords:** Oncology, Diagnostic imaging

## Abstract

**BACKGROUND:**

Considering that trophoblast cell-surface antigen 2 (Trop2) is overexpressed in a wide range of human epithelial cancers, it presents an attractive target for diagnosis and treatment of multiple types of cancer. Herein, we have developed a Trop2-specific radiotracer, ^68^Ga-MY6349, and present a prospective, investigator-initiated trial to explore the clinical value of ^68^Ga-MY6349 PET/CT.

**METHODS:**

In this translational study, 90 patients with 15 types of cancer who underwent ^68^Ga-MY6349 PET/CT were enrolled prospectively. Among them, 78 patients underwent paired ^68^Ga-MY6349 and ^18^F-FDG PET/CT, and 12 patients with prostate cancer underwent paired ^68^Ga-MY6349 and ^68^Ga-PSMA-11 PET/CT.

**RESULTS:**

Among the 90 patients across 15 types of cancer, ^68^Ga-MY6349 uptake in tumors was generally high but heterogeneous, varying among lesions, patients, and cancer types. Trop2 expression level determined by immunohistochemistry was highly correlated with ^68^Ga-MY6349 uptake at primary and metastatic tumor sites. ^68^Ga-MY6349 PET/CT showed higher tumor uptake (quantified by maximum standardized uptake value) than ^18^F-FDG PET/CT in certain types of cancer, including breast (7.2 vs. 5.4, *P* < 0.001), prostate (9.2 vs. 3.0, *P* < 0.001), and thyroid cancers (8.5 vs. 3.7, *P* < 0.001). Compared with ^68^Ga-PSMA-11, ^68^Ga-MY6349 PET/CT exhibited comparable lesion uptake (12.2 vs. 12.5, *P* = 0.223) but a better tumor-to-background contrast (15.8 vs. 12.2, *P* < 0.001) for primary and metastatic prostate cancer, allowing visualization of more metastatic lesions.

**CONCLUSION:**

^68^Ga-MY6349 PET/CT is a noninvasive method for comprehensively assessing Trop2 expression in tumors, which can improve diagnosis and staging for cancer patients and aid in decision making for Trop2-targeted therapies and advancing of personalized treatment.

**TRIAL REGISTRATION:**

ClinicalTrials.gov NCT06188468.

**FUNDING:**

National Natural Science Foundation of China, National Key R&D Program of China, Nuclear Energy R&D project, Fujian Research and Training Grants for Young and Middle-aged Leaders in Healthcare, Key Scientific Research Program for Young Scholars in Fujian, and Fujian Natural Science Foundation for Distinguished Young Scholars.

## Abstract 2

Trop2-targeted molecular imaging in 15 types of cancers

## Graphical abstract

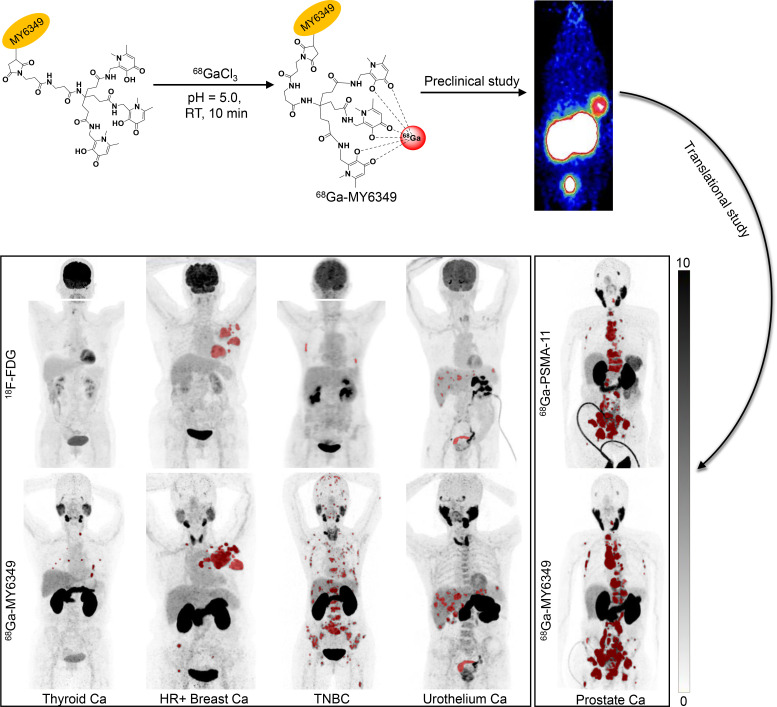

## Introduction

Trophoblast cell-surface antigen 2 (Trop2), a transmembrane glycoprotein, exhibits high expression in most epithelial cancers and low expression in most normal tissues (1–3). Elevated Trop2 expression is associated with increased tumor cell proliferation and invasion (4), and is correlated with poor prognosis in various cancers (5–7). Thus, Trop2 could be a crucial target for clinical diagnosis and therapeutic intervention in a range of tumors.

Sacituzumab govitecan (SG), an antibody-drug conjugate (ADC) that targets Trop2, has gained US Food and Drug Administration (FDA) approval for use in treating metastatic triple-negative breast cancer (TNBC), hormone receptor–positive breast cancer (HR^+^ BC), and metastatic urothelial carcinoma (5, 8). Datopotamab deruxtecan, another Trop2-targeting ADC, recently demonstrated encouraging progression-free survival in the phase III TROPION-Lung01 trial for advanced non-squamous non–small cell lung cancer (NSCLC) (9). The phase III ASCENT study (SG versus chemotherapy for metastatic TNBC) suggested that SG efficacy was closely related to Trop2 expression level (10). These data highlight the need for accurate and comprehensive evaluation of Trop2 expression in tumors to stratify patients for precise treatment (11).

The current method for detecting Trop2, based on the immunohistochemical (IHC) staining of biopsy specimens, has limitations due to the temporal and spatial heterogeneity of Trop2 expression in tumors. Noninvasive nuclear medical imaging may overcome these shortcomings and detect changes of Trop2 expression during treatment to guide subsequent therapies.

In this study, we developed a Trop2-specific radiotracer, ^68^Ga-MY6439, for PET/CT imaging and clinical translation in various cancer types. Although ^18^F-FDG PET/CT is a standard diagnostic method for tumor imaging in clinical nuclear medicine, it has limitations, including diagnostic sensitivity and specificity. Furthermore, ^18^F-FDG PET/CT cannot provide information about Trop2 expression in tumors. Therefore, we compared the imaging diagnostic characteristics of ^68^Ga-MY6349 PET/CT and ^18^F-FDG PET/CT in different tumors to explore whether ^68^Ga-MY6349 PET/CT could compensate for some of these shortcomings. Our primary objective was to assess the feasibility of ^68^Ga-MY6349 PET/CT imaging for the noninvasive assessment of Trop2 expression in various cancer types, promoting precision therapy of Trop2-targeted ADCs, and expand future application. Our secondary objective was to investigate the superiority of ^68^Ga-MY6349 PET/CT over ^18^F-FDG PET/CT in some tumors through comparative analysis, providing valuable insights for cancer diagnosis and staging.

## Results

### Preparation and preclinical evaluation of ^68^Ga-MY6439.

**Figure 1:**
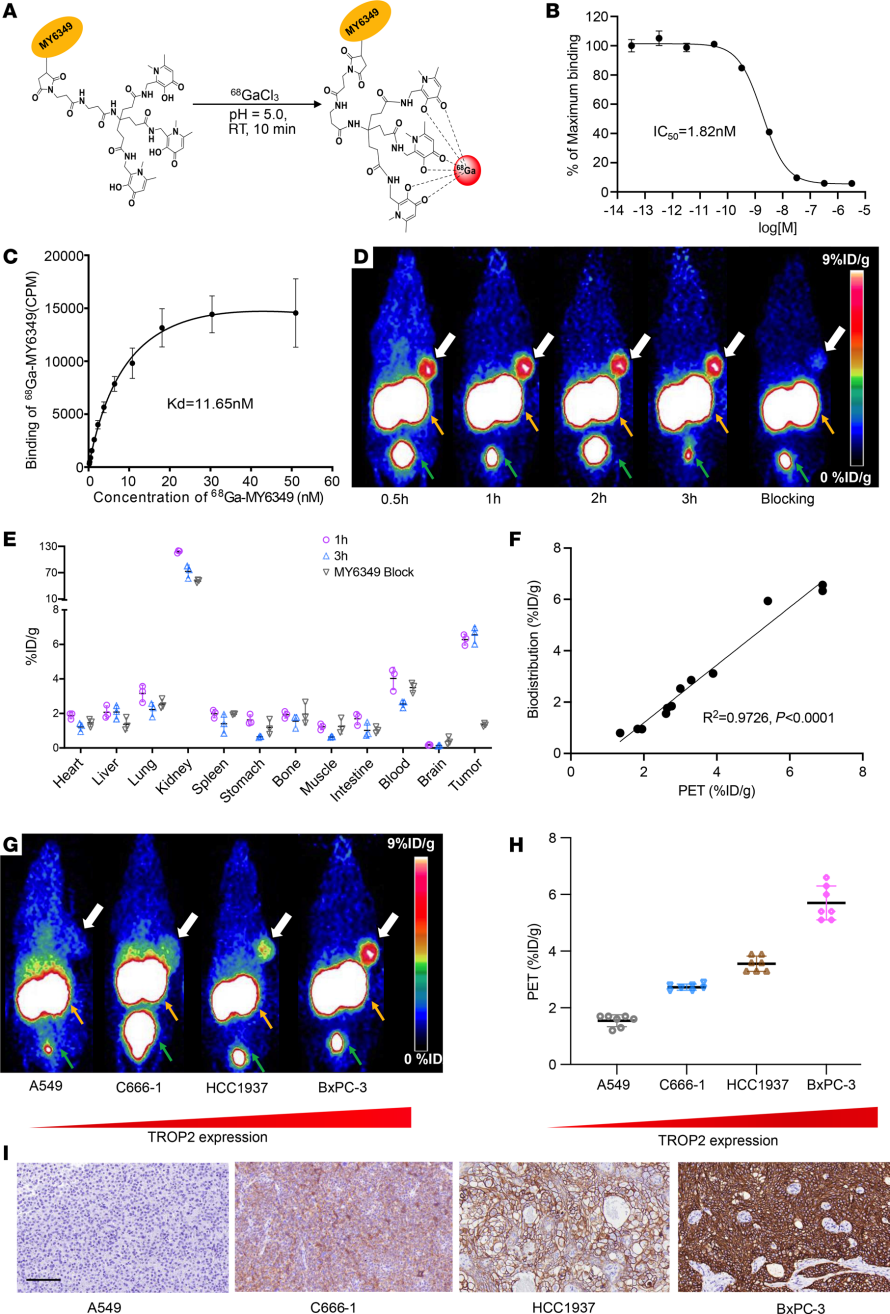
Preparation and preclinical evaluation of ^68^Ga-MY6439. (**A**) The mechanism for radiolabeling MY6349 with ^68^Ga. RT, room temperature. (**B** and **C**) The binding affinity of ^68^Ga-MY6349 to Trop2, analyzed through half-maximal inhibitory concentration and equilibrium dissociation constant. (**D**) Representative PET images of ^68^Ga-MY6349 and blocking experiment in the BxPC-3 tumor model. Yellow arrows, kidneys; green arrows, bladder; white arros, tumor. (**E**) Quantification results from biodistribution studies of ^68^Ga-MY6349 with or without the simultaneous injection of unlabeled MY6349. (**F**) The correlation between PET region of interest (ROI) and biodistribution studies (%ID/g). (**G** and **H**) Representative PET images and quantification results of nude mice bearing A549, C666-1, HCC1937, and BxPC-3 tumors at 1 hour after injection, demonstrating Trop2-specific tumoral uptake. The second image in **D** is shown again as the last image in **G**. Yellow arrows, kidneys; green arrows, bladder. (**I**) IHC staining for Trop2 in the corresponding tumors. Scale bar: 100 μm.

The Trop2-targeted nanobody MY6349 was modified and radiolabeled with ^68^Ga, achieving radiochemical purity greater than 95% and specific radioactivity of 146 GBq/μmol (Figure 1A). Stability tests indicated that ^68^Ga-MY6349 remained stable in PBS and FBS for up to 4 hours, ensuring its suitability for further studies without additional purification (Supplemental Figure 1; supplemental material available online with this article; https://doi.org/10.1172/JCI185408DS1). Regarding the Trop2 expression in the cell lines, BxPC-3 cells had the highest Trop2 expression, followed by HCC1937 and C666-1, with A549 showing negligible expression (Supplemental Figure 2A). Moreover, the colocalization of FITC-MY6349 and full-length Trop2 antibody, observed through flow cytometry and confocal microscopy, validated the specificity of MY6349 to Trop2 (Supplemental Figure 2, B and C). ^68^Ga-MY6349 exhibited efficient binding to Trop2-positive cell lines, and binding specificity was confirmed by marked binding inhibition in the presence of unlabeled MY6349 and full-length Trop2 antibody, as well as negligible binding to Trop2-negative A549 cells (Supplemental Figure 3). The binding assays demonstrated a high affinity of ^68^Ga-MY6349 for Trop2, with a 50% inhibitory concentration (IC_50_) of 1.82 nM and an equilibrium dissociation constant of 11.65 nM (Figure 1, B and C).

Small-animal PET imaging in BxPC-3 tumor xenografts showed rapid tumor accumulation of ^68^Ga-MY6349 at 0.5 hours after injection (6.3 ± 0.5 percent of the injected dose per gram of tissue [%ID/g]), maintaining sustained level up to 3 hours after injection (6.3 ± 0.8 %ID/g). Coadministration with MY6349 substantially reduced tumor uptake, reconfirming the specificity of ^68^Ga-MY6349 (Figure 1D). Ex vivo biodistribution studies corroborated these findings (Figure 1E), and the semiquantitative PET data exhibited a strong correlation with the quantitative biodistribution (*R*^2^ = 0.9726, *P* < 0.0001) (Figure 1F). The IHC results were in line with ^68^Ga-MY6349 PET findings (Figure 1, G–I). These results underscore the capability of ^68^Ga-MY6349 PET/CT to accurately reflect Trop2 expression in vivo.

### Patient characteristics, safety evaluation, and in vivo biodistribution pattern of ^68^Ga-MY6349.

**Figure 2:**
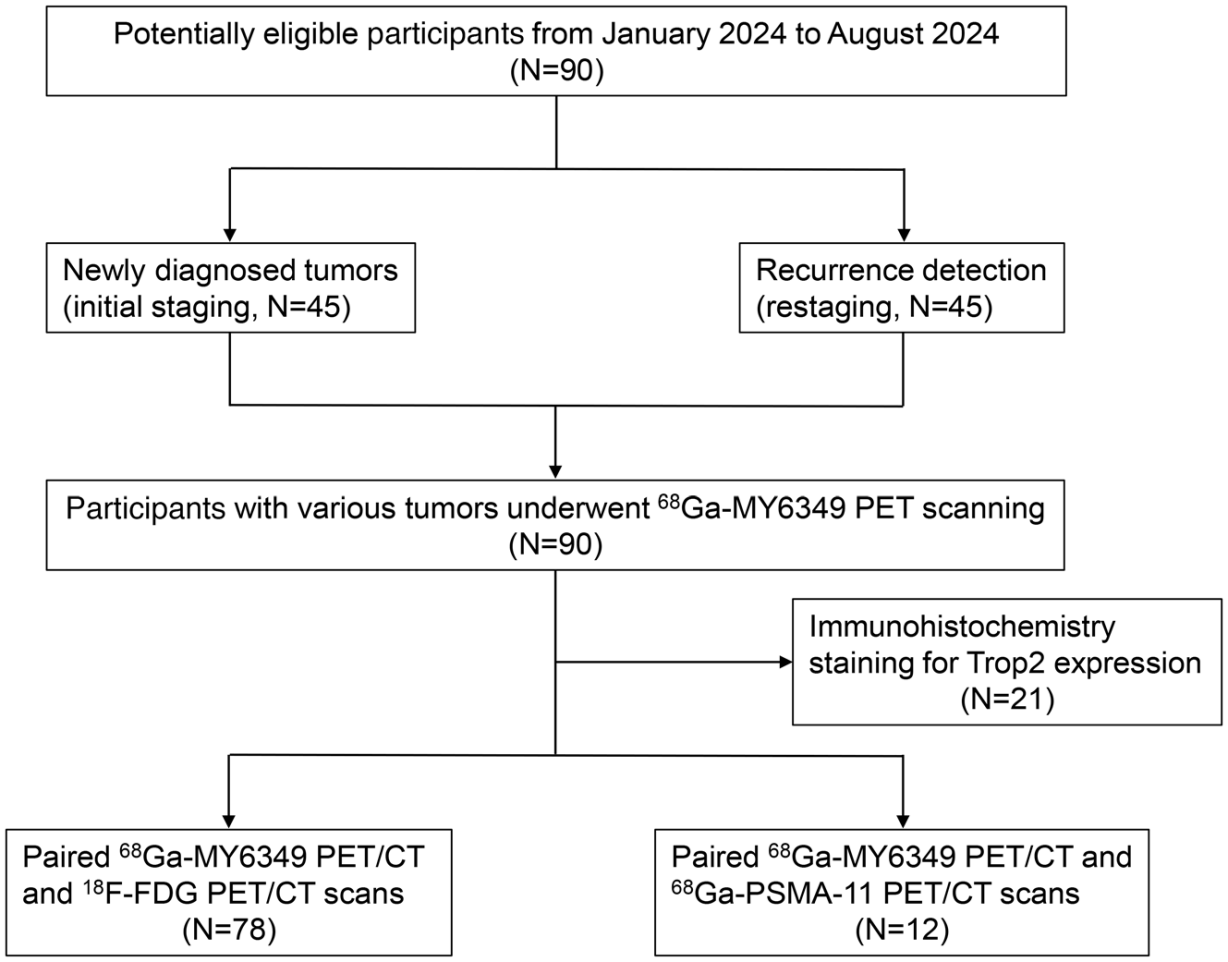
Flow diagram outlining the clinical study design and participating patients.

In this clinical translational study conducted between January 2024 and August 2024, 90 patients with 15 cancer types who underwent ^68^Ga-MY6349 PET/CT were prospectively enrolled (Figure 2). The patient clinical characteristics are shown in Supplemental Table 1. Among the 90 patients, 78 patients underwent paired ^68^Ga-MY6349 PET/CT and ^18^F-FDG PET/CT scans. Additionally, 12 patients with prostate cancer underwent paired ^68^Ga-MY6349 PET/CT and ^68^Ga-PSMA-11 PET/CT (the standard-of-care imaging for prostate cancer). In this cohort, 45 treatment-naive patients underwent PET/CT for initial tumor staging, and the other 45 patients underwent PET/CT for the identification of tumor recurrence and metastases. Diagnoses were confirmed via pathology (from biopsy or surgery samples) in 67 patients and via imaging and/or clinical follow-up in the remaining 23 patients.

All participants tolerated ^68^Ga-MY6349 well, with no adverse events reported during injection or the 4-hour follow-up period. Supplemental Figure 4 shows representative PET-MIP (maximum-intensity projection) images at various time points along with biodistribution data in normal organs (for 3 patients). The effective dose of ^68^Ga-MY6349, calculated using OLINDA/EXM software, was 1.46 × 10^−2^ mSv/MBq (Supplemental Table 2). As the tracer uptake in the blood pool diminished over time, the image at 60 minutes after injection provided a favorable tumor-to-background ratio (TBR). Therefore, 60 minutes was selected as the optimal time for subsequent imaging studies. The in vivo distribution pattern of ^68^Ga-MY6349 was primarily observed in the kidneys, pancreas, salivary glands, and thyroid gland (Supplemental Figure 4B).

### ^68^Ga-MY6349 PET/CT imaging in 15 types of cancer.

**Figure 3:**
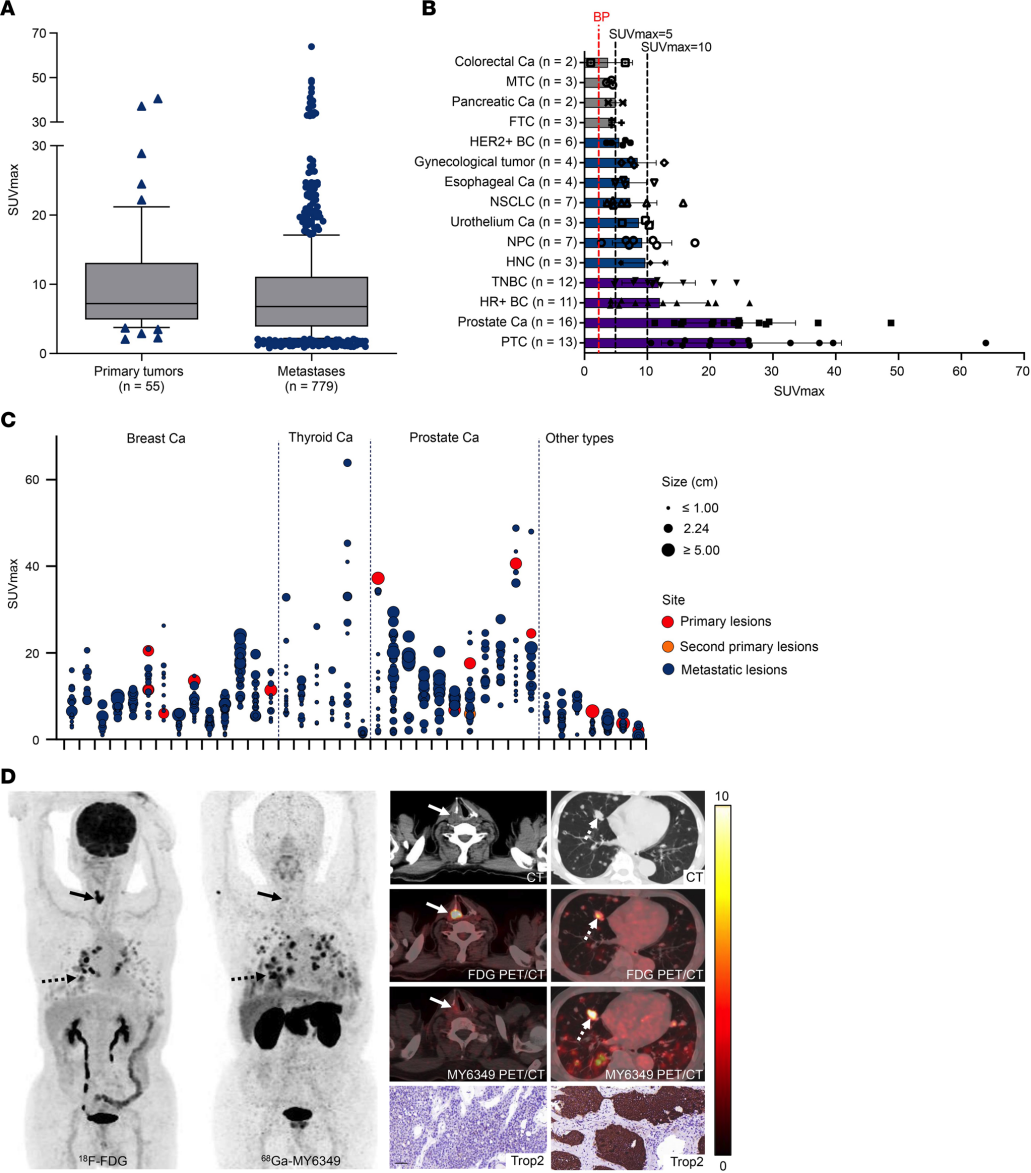
^68^Ga-MY6349 uptake across various cancers. (**A**) There were no significant differences in SUVmax values derived from ^68^Ga-MY6349 PET/CT between primary and metastatic tumors. (**B**) The average SUVmax values were derived from ^68^Ga-MY6349 PET/CT across 15 tumor entities, showing a distribution of low, intermediate, and high uptake, defined by cutoff values at SUVs of 5 and 10, respectively. Conversely, the average background (blood pool) SUV was 2.1. Ca, cancer; FTC, follicular thyroid carcinoma; HER2^+^ BC, human epidermal growth factor receptor 2–positive breast cancer; HNC, head and neck cancer; HR^+^ BC, hormone receptor–positive breast cancer; MTC, medullary thyroid cancer; NPC, nasopharyngeal carcinoma; NSCLC, non–small cell lung cancer; PTC, papillary thyroid carcinoma; TNBC, triple-negative breast cancer. (**C**) Overview of ^68^Ga-MY6349 uptake as SUVmax in 38 patients, including 15 primary, 1 second primary, and 665 metastatic lesions. The SUVmax for different tumor lesions within the same patient is also shown, visualizing tumor size and site. (**D**) PET/CT images of a patient (metastatic PTC) illustrate different ^68^Ga-MY6349 uptake between the recurrent tumor (solid arrows) and metastatic lung lesions (dotted arrows). ^18^F-FDG and ^68^Ga-MY6349 PET/CT scans were performed in the same patient. Scale bar: 100 μm.

Fifty-five primary tumors (7 patients with multiple primary tumors) and 779 recurrences/metastases were identified. The comparison of ^68^Ga-MY6349 uptake between primary and metastatic lesions indicated no significant disparity (*P* = 0.07; Figure 3A), with an average maximum standardized uptake value (SUVmax) of 7.1 for primary lesions (interquartile range [IQR] 4.8–13.0) and 6.8 for metastatic lesions (IQR 3.9–11.1). Further analyses of ^68^Ga-MY6349 uptake across various tumor varieties (Figure 3B) showed that the tumor types with higher ^68^Ga-MY6349 uptake (average SUVmax > 10) were TNBC, HR^+^ BC, prostate cancer, and papillary thyroid carcinoma (PTC). Moderate uptake levels (average SUVmax 5–10) were observed in human epidermal growth factor receptor 2–positive breast cancer (HER2^+^ BC), gynecological tumors, esophageal cancer, NSCLC, urothelial cancer, nasopharyngeal carcinoma (NPC), and head and neck cancer. Lower ^68^Ga-MY6349 uptake (mean SUVmax < 5) was observed in colorectal cancer, medullary thyroid cancer, pancreatic cancer, and follicular thyroid carcinoma. To evaluate whether the heterogeneity of Trop2 expression in tumors could be visualized via Trop2-targeted PET/CT, we analyzed ^68^Ga-MY6349 tumor uptake in different lesions within the same patient (Figure 3C). The within-patient SUVmax heterogeneity was observed in 38 patients, each with more than 10 lesions. The median fold difference was 4.5 (range 1.8–20.6), and the median coefficient of variation was 38.9% (range 19.1%–84.0%). The representative case reflecting the within-patient heterogeneity of Trop2 expression is presented in Figure 3D, showing different ^68^Ga-MY6349 uptake between the recurrent tumor (negative Trop2 expression, SUVmax 1.7) and metastatic lung lesions (positive Trop2 expression, SUVmax 13.7) in a patient with metastatic thyroid cancer. Figure 3D also shows that ^18^F-FDG PET/CT can detect metastatic tumors, but cannot reflect the Trop2 expression in tumors.

**Figure 4:**
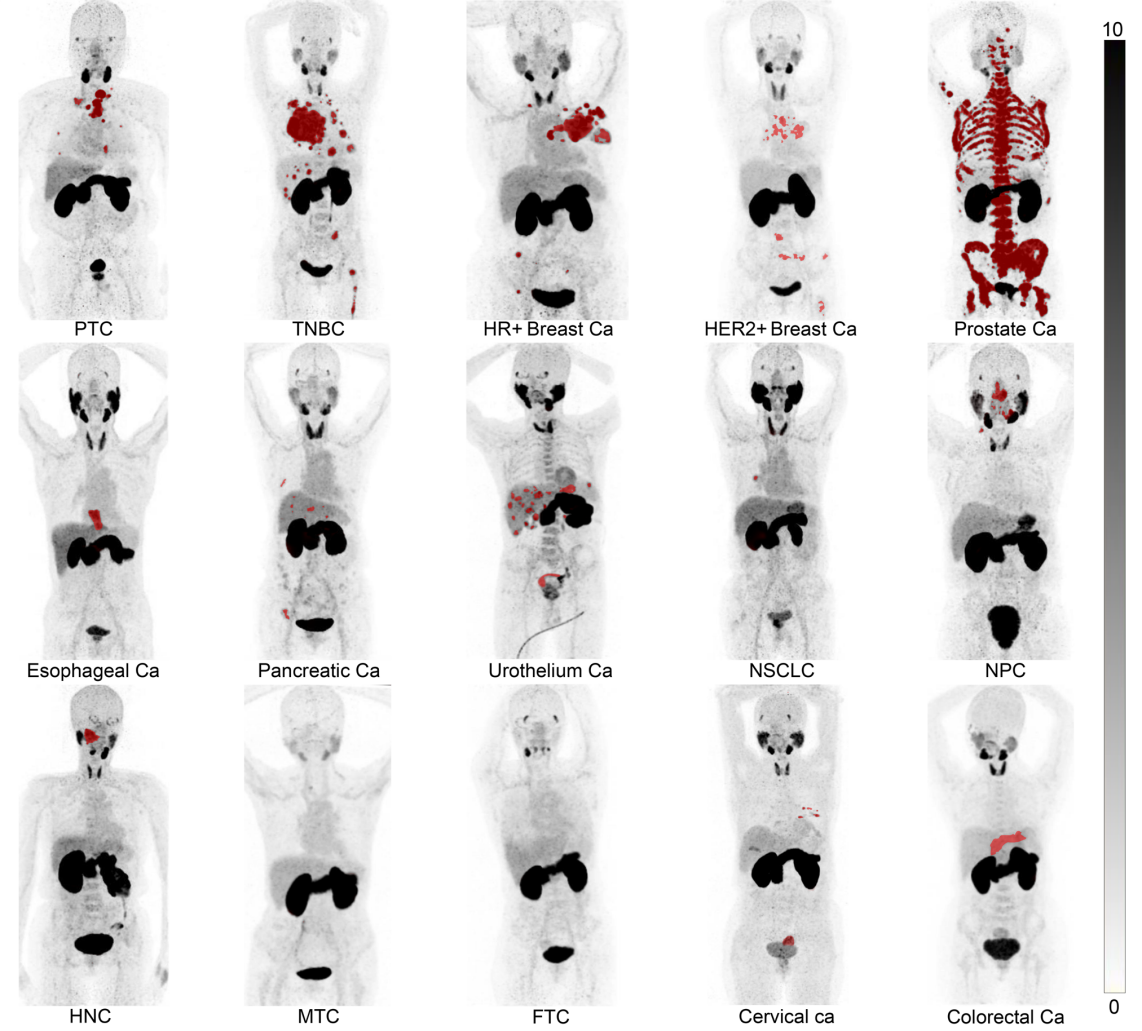
^68^Ga-MY6349 PET/CT in patients with 15 different histologically confirmed tumor entities. Ca, cancer; FTC, follicular thyroid carcinoma; HER2, human epidermal growth factor receptor 2; HNC, head and neck cancer; HR, hormone receptor; MTC, medullary thyroid cancer; NPC, nasopharyngeal carcinoma; NSCLC, non–small cell lung cancer; PTC, papillary thyroid carcinoma; TNBC, triple-negative breast cancer.

The swift clearance of ^68^Ga-MY6349 from the blood pool contributed to the low background activity 1 hour after injection, with a mean SUVmax of 2.1 for the blood pool and 0.7 for muscle tissues. This clearance resulted in a TBR greater than 3 for most tumor types, enabling clear delineation of tumor contours to improve PET/CT diagnostic accuracy (Figure 4).

**Figure 5:**
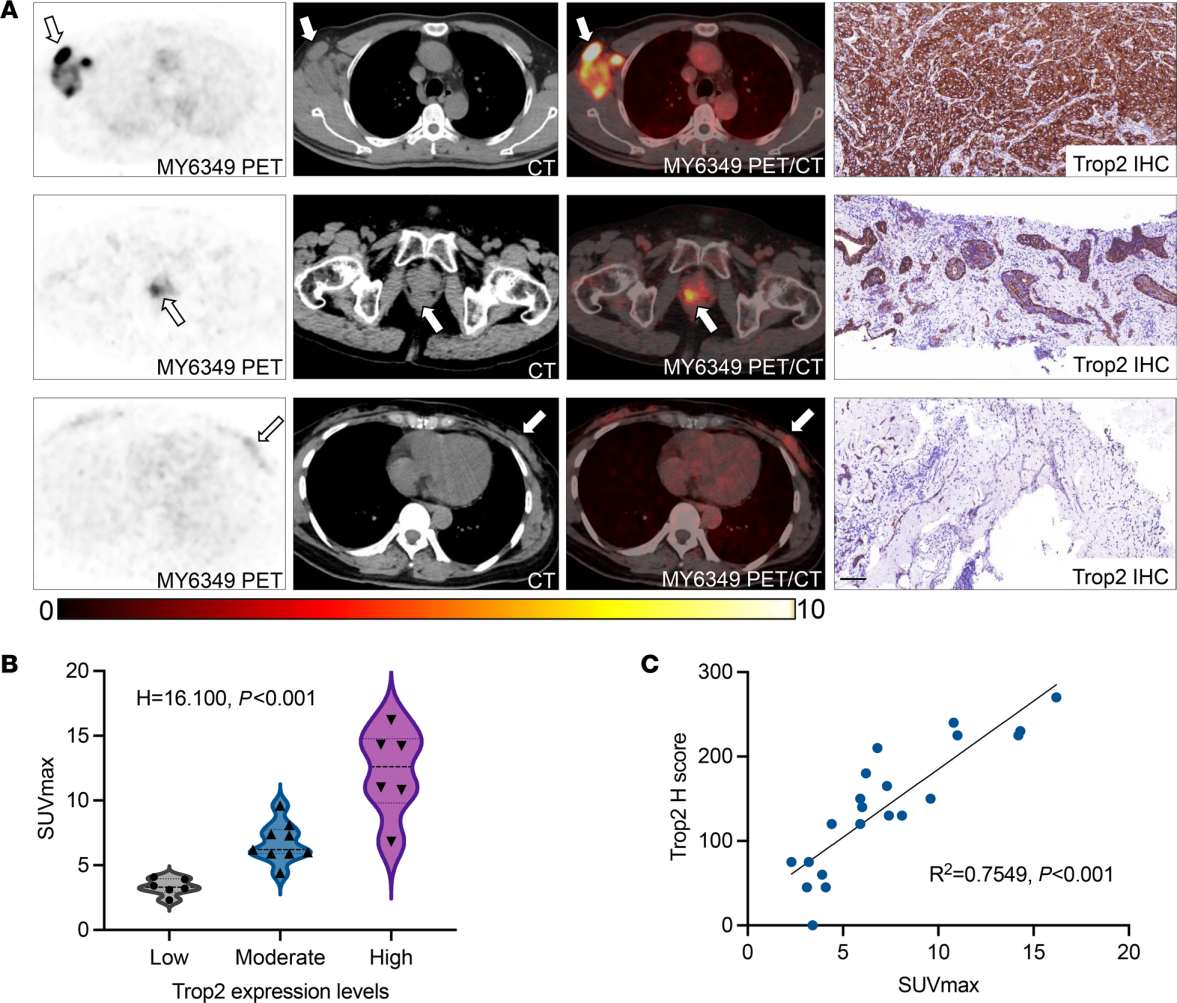
The correlation between ^68^Ga-MY6349 tumor uptake and Trop2 expression levels. (**A**) Representative ^68^Ga-MY6349 PET/CT images and immunohistochemically stained tissue samples from patients with various tumor types and different Trop2 expression scores (white arrows indicate tumor lesions). Top row, newly diagnosed TNBC; middle row, newly diagnosed prostate cancer; bottom row, newly diagnosed HER2^+^ breast cancer. Different levels of ^68^Ga-MY6349 uptake were observed in different tumor lesions with different Trop2 expression levels. First, SUVmax 16.2, Trop2 H score 270; second, SUVmax 9.6, Trop2 score 150; third, SUVmax 4.1, Trop2 score 45. (**B**) A significant correlation between SUVmax and Trop2 expression was determined using the Kruskal-Wallis test (H = 16.100, *P* < 0.001). (**C**) The correlation between SUVmax and Trop2 H scores based on simple linear regression (*R*^2^ = 0.7549, *P* < 0.001). Scale bar: 100 μm.

We analyzed 21 eligible biopsy specimens, comprising 12 primary tumors and 9 metastatic lesions, for further Trop2 IHC staining. Trop2 expression was evaluated based on the H score methodology used in the phase III ASCENT trial. Among the specimens, 6 displayed low Trop2 expression (H score < 100), 9 showed medium Trop2 expression (H score 100–200), and 6 exhibited high Trop2 expression (H score 200–300). A positive correlation was identified between ^68^Ga-MY6349 tumor uptake and Trop2 expression levels (*P* < 0.001; Figure 5, A and B). Furthermore, a linear relationship was observed between the 2 variables, with a coefficient of determination (*R*^2^) of 0.7549 and *P* less than 0.001 (Figure 5C).

We also conducted ^68^Ga-MY6349 PET/CT scans before and 24 hours after SG administration in 2 patients to evaluate the occupation of Trop2 target sites. As depicted in Supplemental Figure 5, the lesions initially exhibited high ^68^Ga-MY6349 uptake at baseline. After treatment, there was a substantial reduction of ^68^Ga-MY6349 uptake across all lesions, suggesting that the Trop2 target sites were effectively bound by the Trop2-targeted SG, which forms the basis for the therapeutic effect of SG.

### ^68^Ga-MY6349 PET/CT demonstrated superiority over ^18^F-FDG PET/CT in diagnosis of certain cancer types.

Because of the remarkably high ^68^Ga-MY6349 uptake in many epithelial tumors and low uptake in normal organs, we further analyzed the diagnostic efficacy of ^68^Ga-MY6349 PET/CT for various cancer types and compared the results with those of ^18^F-FDG PET/CT, the most commonly used method in routine clinical practice. For the diagnosis of primary tumors, there was no statistical difference between ^68^Ga-MY6349 uptake and ^18^F-FDG uptake in NPC, HR^+^ BC, HER2^+^ BC, TNBC, esophageal cancer, NSCLC, gynecological tumors, and prostate cancer (Table 1). However, ^68^Ga-MY6349 PET/CT showed a superior TBR compared with that of ^18^F-FDG PET/CT in HR^+^ breast cancer (11.0 vs. 4.0, *P* = 0.002), leading to the detection of more breast tumor lesions (14 vs. 12). Similar results were observed in HER2^+^ BC, urothelial cancer, and prostate cancer, in which ^68^Ga-MY6349 PET/CT showed a greater TBR and more tumor lesions than did ^18^F-FDG PET/CT (Table 1).

**Figure 6:**
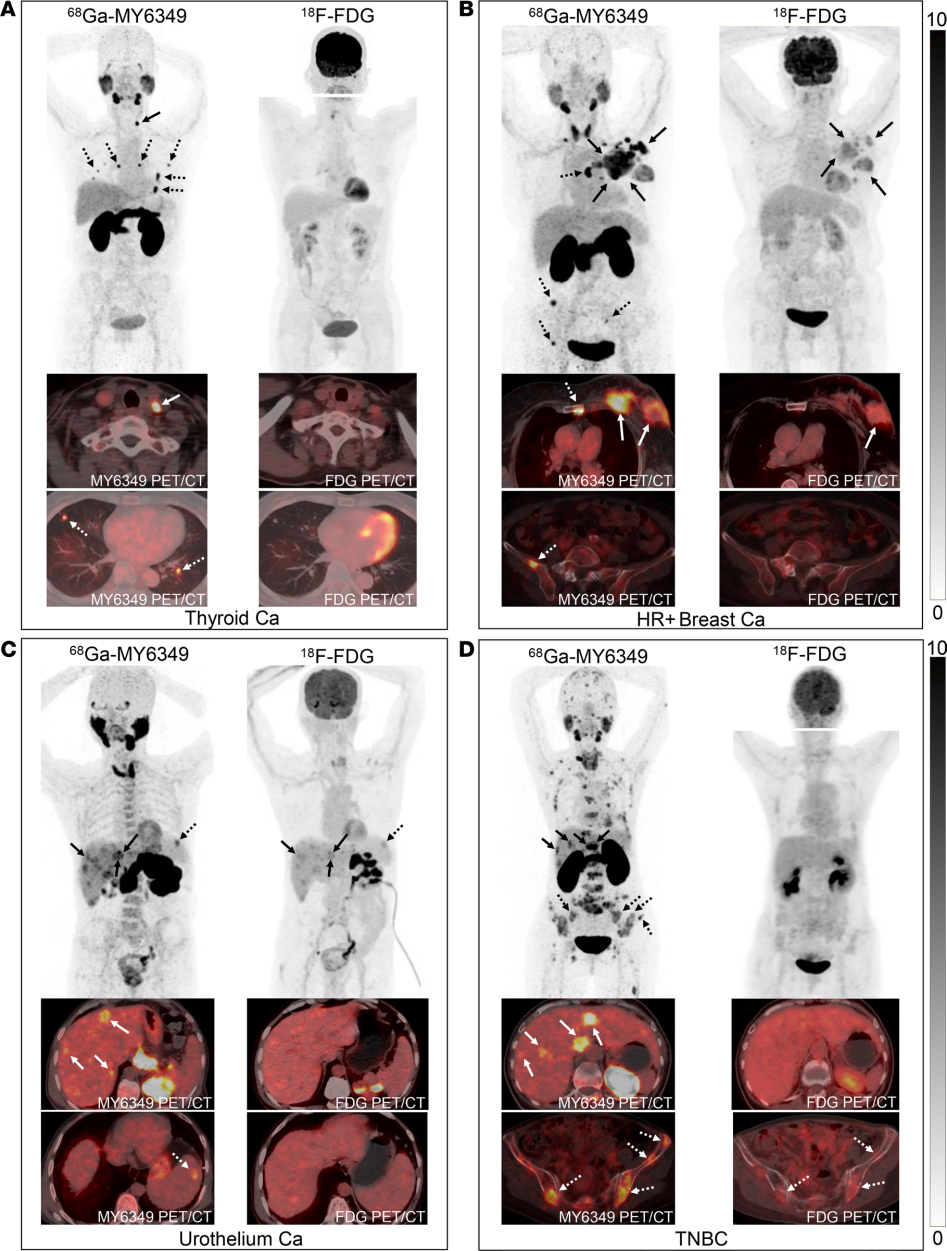
Representative ^18^F-FDG PET/CT and ^68^Ga-MY6349 PET/CT images in patients with various cancers. (**A**) A 68-year-old woman with papillary thyroid carcinoma had undergone surgical resection 1 year prior; PET/CT was performed to detect tumor recurrence. ^68^Ga-MY6349 PET/CT showed intense uptake in the neck lymph node (solid arrows) and lung metastases (dotted arrows), but ^18^F-FDG PET/CT demonstrated false-negative findings. (**B**) A 74-year-old woman with HR^+^ breast cancer underwent PET/CT for tumor staging. ^68^Ga-MY6349 PET/CT showed higher radiotracer uptake than that of ^18^F-FDG PET/CT in the primary tumor (solid arrows), lymph node, and bone metastases (dotted arrows). Additionally, ^18^F-FDG PET/CT showed false-negative uptake in the bone metastases. The HR^+^ breast cancer image from Figure 4 is shown again in **B**. (**C**) A 63-year-old man with urothelial cancer underwent PET/CT for initial staging. ^68^Ga-MY6349 PET/CT demonstrated higher tracer uptake and better image contrast than ^18^F-FDG PET/CT in the primary and metastatic lesions, particularly for the liver metastases (solid arrows) and spleen metastasis (dotted arrows). The urothelium cancer image from Figure 4 is shown again in **C**. (**D**) A 65-year-old woman with previously treated breast cancer (TNBC) underwent PET/CT for restaging. ^68^Ga-MY6349 PET/CT showed intense tracer uptake in the lymph nodes, liver (solid arrows), and widespread bone metastases (dotted arrows). However, ^18^F-FDG PET/CT showed low to mild uptake in most of the metastases.

Regarding the detection of recurrent and metastatic tumors (Table 2), ^68^Ga-MY6349 PET/CT had a higher SUVmax and TBR than ^18^F-FDG PET/CT in PTC (Supplemental Table 3), prostate cancer, and HR^+^ breast cancer, thus identifying more metastatic lesions, particularly for the detection of lymph node metastases (e.g., PTC), bone metastases (e.g., HR^+^ breast cancer), and liver metastases (e.g., urothelial carcinoma). Representative cases are presented in Figure 6, A–C. Intriguingly, although the SUVmax derived from ^68^Ga-MY6349 PET/CT was similar to that of ^18^F-FDG PET/CT in TNBC lesions, the TBR was considerably higher, facilitating the detection of additional occult or small lesions (135 vs. 123), particularly for liver and bone metastases (Figure 6D). Conversely, ^18^F-FDG PET/CT exhibited a higher SUVmax and allowed for the detection of more metastatic lesions in NPC, HER2^+^ BC, esophageal cancer, pancreatic cancer, and gynecological tumors. The findings underscore the potential of ^68^Ga-MY6349 PET/CT as either a complementary or alternative imaging approach for certain cancer types, enhancing neoplasm detection and characterization.

### Comparison of ^68^Ga-MY6349 PET/CT and ^68^Ga-PSMA-11 PET/CT in prostate cancer.

**Figure 7:**
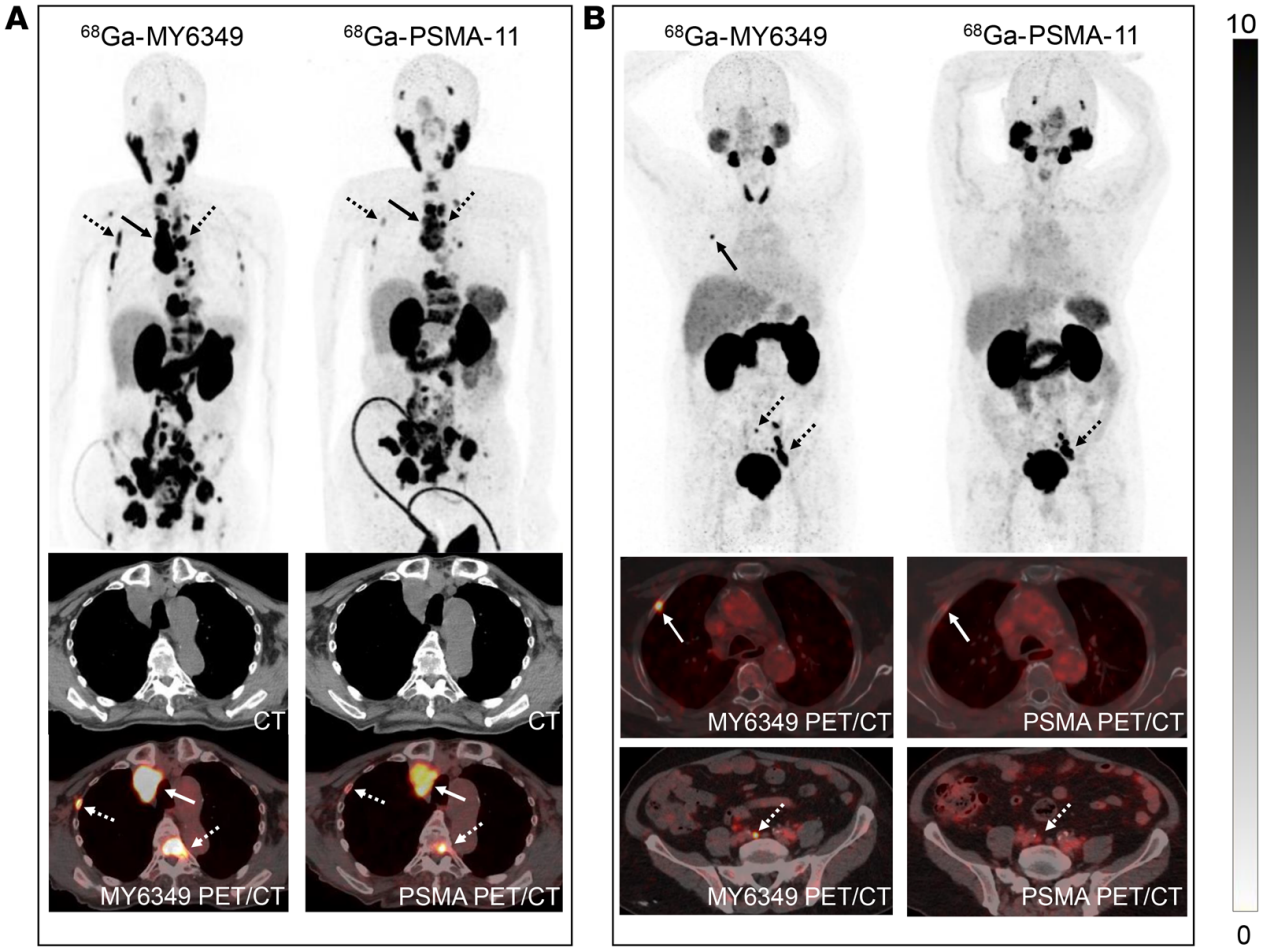
Representative ^68^Ga-MY6349 PET/CT and ^68^Ga-PSMA-11 PET/CT images in patients with metastatic prostate cancer. (**A**) A 77-year-old man with prostate cancer who had received endocrine therapy for 3 years underwent PET/CT imaging for restaging. Compared with ^68^Ga-PSMA-11 PET/CT, ^68^Ga-MY6349 PET/CT revealed more metastatic lesions and exhibited higher tracer uptake in the lymph node metastasis (solid arrows) and bone metastases (dotted arrows). (**B**) A 70-year-old man with pathologically confirmed prostate cancer underwent PET/CT for initial staging. ^68^Ga-MY6349 PET/CT demonstrated higher tracer uptake in the bone metastasis (solid arrows) and the involved lymph node (dotted arrows) than that of 68Ga-PSMA-11 PET/CT.

We compared ^68^Ga-MY6349 with another FDA-approved PET tracer, ^68^Ga-PSMA-11, for the diagnosis of prostate cancer. Twelve patients with confirmed prostate cancer underwent paired ^68^Ga-MY6349 PET/CT and ^68^Ga-PSMA-11 PET/CT, which revealed no difference in radiotracer uptake between the primary and recurrent tumors. However, ^68^Ga-MY6349 PET/CT exhibited a superior TBR in metastatic lesions compared with that of ^68^Ga-PSMA-11 PET/CT, leading to the detection of more bone metastases (122 vs. 101; Supplemental Table 4) and comparable lymph node metastases (61 vs. 60). The representative cases showing the superiority of ^68^Ga-MY6349 PET/CT over ^68^Ga-PSMA-11 PET/CT are presented in Figure 7.

## Discussion

Trop2 is a pivotal biomarker in tumor research owing to its overexpression in most solid epithelial tumors (1–3). A macroscopic, noninvasive molecular imaging technique to assess Trop2 expression in tumors could offer novel insights into cancer diagnosis and therapy. Notably, clinical trials have demonstrated the efficacy of Trop2-targeted ADCs, including SG, in treating breast cancer (TNBC and HR^+^ breast cancer) and urothelial cancer, leading to FDA approval of SG for these two cancer types (12–14). Nuclear medicine molecular imaging with a Trop2-specific radiotracer could be instrumental in identifying patients likely to benefit from SG treatment, and broadening the potential indications for future applications of Trop2-targeted ADCs. Given the high and specific Trop2 expression in various solid epithelial tumors, ^68^Ga-MY6349 PET/CT should have substantial clinical value to be explored.

The preclinical results indicated that ^68^Ga-MY6439 possessed high specificity and binding affinity for the Trop2 receptor. PET imaging and biodistribution data across various tumor xenografts underscored the capability of ^68^Ga-MY6349 PET/CT to differentiate Trop2 expression levels in vivo. In comparison with preclinical studies that used radiolabeled monoclonal antibodies as PET/single-photon emission CT (SPECT) tracers for Trop2 imaging, which typically requires 24–48 hours to achieve optimal tumor uptake (15–17), the rapid tumor accumulation and fast blood clearance rate of ^68^Ga-MY6349 resulted in favorable tumor-to-background contrast within 1 hour after tracer injection. This enables patients to complete the entire PET/CT imaging workflow within one day, substantially improving patient compliance and reducing radiation exposure. In another series of Trop2-specific nanobody tracers (^68^Ga-NOTA-RTD98, ^68^Ga-NOTA-RTD161, ^68^Ga-NOTA-RTD01, and ^68^Ga-NOTA-T4) that were recently developed by Huang et al. (18, 19), ^68^Ga-MY6349 demonstrated the lowest liver uptake and a favorable tumor-to-liver ratio in mice bearing BxPC-3 tumors. Moreover, ^68^Ga-MY6349 exhibited higher tumor uptake than that of ^68^Ga-NOTA-T4 (6.3 ± 0.3 vs. 4.6 ± 1.4 %ID/g, 45–60 minutes after injection). In Huang’s translational study, 10 patients underwent ^68^Ga-NOTA-T4 PET/CT, with only 3 receiving paired ^18^F-FDG and ^68^Ga-NOTA-T4 PET/CT scans (19). Different from the previous foundational literature, we conducted a systematic and in-depth clinical investigation that included a head-to-head comparison of ^18^F-FDG and ^68^Ga-MY6349 PET/CT in 90 patients with various cancer types. We also completed dosimetry analysis and assessed clinical consistency between tumor uptake and Trop2 IHC results. Furthermore, we demonstrated the tumor types that were most suitable for PET/CT imaging with ^68^Ga-MY6349 and presented the potential clinical indications for the future use of Trop2-targeted PET/CT, along with a quantitative analysis of lesions in patients with breast cancer before and after treatment with Trop2-targeted ADC drugs. This work contributes to establishing an imaging method to improve the diagnosis and staging of cancer patients as well as future clinical application of Trop2-targeted therapies, including ADC therapies, toward precision theranostics.

We first evaluated the safety, dosimetry, and in vivo distribution pattern of ^68^Ga-MY6349 in 3 patients. According to PET/CT imaging, the higher physiological uptake of ^68^Ga-MY6349 was observed in the kidneys, pancreas, and salivary glands, while other organs displayed exceptionally low uptake. The high ^68^Ga-MY6349 uptake in the pancreas and salivary glands was attributed to innate Trop2 expression (20). The high uptake in the kidneys indicated that ^68^Ga-MY6349 was excreted through the urinary system. To our knowledge, this is the first imaging agent targeting Trop2 for which a human dosimetry study has been accomplished. The effective dose of ^68^Ga-MY6349 (1.46 × 10^−2^ mSv/MBq) was comparable to that of other FDA-approved radiopharmaceuticals, including ^18^F-FDG (1.53 × 10^−2^ mSv/MBq) (21), ^68^Ga-PSMA-11 (1.47 × 10^−2^ mSv/MBq) (22), and ^68^Ga-DOTATATE (2.10 × 10^−2^ mSv/MBq) (23). Thus, ^68^Ga-MY6349 PET/CT is a safe imaging method with a low radiation dose.

Based on the high Trop2 expression in many epithelial tumors and low expression in normal tissues, Trop2-specific radiotracers may serve as a new pan-cancer imaging agent class. To validate our hypothesis, we performed ^68^Ga-MY6349 PET/CT imaging in 90 patients with 15 types of cancer. Moderate to high tumor uptake of ^68^Ga-MY6349 was demonstrated, with a favorable TBR in multiple tumor types without significant differences between primary and metastatic tumors. This underscores the potential of ^68^Ga-MY6349 PET/CT for comprehensive tumor imaging. Moreover, we observed a linear correlation between ^68^Ga-MY6349 uptake in various tumor entities and Trop2 IHC scores. This suggests that ^68^Ga-MY6349 PET/CT could serve as a non-invasive imaging method for assessing Trop2 expression in vivo, aiding in decision-making for Trop2-targeted therapy and advancing personalized treatment. The heterogeneous uptake of ^68^Ga-MY6349 was observed in tumor lesions, varying among lesions, patients, and tumor types. Therefore, ^68^Ga-MY6349 PET/CT can provide a comprehensive assessment of Trop2 expression across all metastatic lesions, offering clear advantages over conventional tissue-based Trop2 expression analyses.

From the semi-quantification analysis across the 15 tumor entities, PTC, prostate cancer, HR^+^ BC, and TNBC exhibited the highest ^68^Ga-MY6349 uptake (average SUVmax > 10), indicating the potential clinical applications of ^68^Ga-MY6349 PET/CT for cancer patients when ^18^F-FDG PET/CT has limitations. For instance, breast cancer sometimes exhibits low to mild tumor uptake during ^18^F-FDG PET/CT, leading to false-negative results in some occult lesions, especially in lymph node and bone metastases (24). Among different subtypes of breast cancers, ^18^F-FDG PET/CT is generally more sensitive in TNBC than in HR^+^ and HER2^+^ breast cancers, as some HR^+^ and HER2^+^ breast tumors cannot be clearly visualized. Therefore, ^18^F-FDG PET/CT has some limitations in the initial staging of breast cancer (25, 26). In addition, osseous metastases from breast cancer frequently exhibit false-negative uptake in ^18^F-FDG PET/CT imaging, with a lower detection rate than that in conventional ^99m^Tc-MDP bone scans (27, 28). PTC is the most common form of differentiated thyroid cancer (DTC). Post-therapeutic iodine-131 (^131^I) whole-body scanning remains indispensable owing to its superior performance in identifying radioiodine-avid DTC lesions. However, 5%–15% of DTC and 50% of metastatic DTC develop into radioactive iodine-refractory DTC (RAIR-DTC), which loses avidity to ^131^I and is prone to aggressiveness (29). In the present study, the majority of patients with DTCs (15/16, 94%) had RAIR-DTCs, for which ^18^F-FDG PET/CT is recommend by the American Thyroid Association guideline for the diagnosis of tumor recurrence and metastases. However, the sensitivity of ^18^F-FDG PET/CT for detecting recurrence/metastasis within the RAIR-DTC varies from 68.8% to 82.0% (30). Furthermore, a false-negative rate of 8.0%–21.1% has been reported in patients with thyroglobulin elevation and negative iodine scintigraphy (31, 32), which further complicates the management of metastatic DTC. Our data indicated that ^68^Ga-MY6349 PET/CT detected more primary and/or metastatic lesions than ^18^F-FDG PET/CT did in prostate cancer, RAIR-DTC, HR^+^ BC, and TNBC. Thus, these tumor types may benefit from ^68^Ga-MY6349 PET/CT imaging. However, in other cancer types, such as NPC, HER2^+^ BC, esophageal cancer, pancreatic cancer, and gynecological tumors, the lesion detectability of ^68^Ga-MY6349 PET/CT was inferior to that of ^18^F-FDG PET/CT. Additionally, compared with ^68^Ga-PSMA-11, the standard-of-care PET tracer for prostate cancer, ^68^Ga-MY6349 PET/CT demonstrated comparable lesion uptake and a better TBR for imaging primary and metastatic lesions, resulting in the visualization of more metastatic lesions, especially for the occult bone metastases. Thus, ^68^Ga-MY6349 PET/CT may offer a novel imaging modality for tumor detection and staging in certain cancer types, improving diagnosis in cancer patients facing limitations in standard-of-care examinations.

Trop2-targeted ADCs have only been approved for advanced breast cancer (TNBC and HR^+^ breast cancer) and urothelial cancer. Here, high ^68^Ga-MY6349 uptake was also found in prostate and thyroid cancer; this may lead to new indications for Trop2-targted ADCs, particularly for metastatic castration-resistant prostate cancer (mCRPC) and RAIR-DTC. Patients with mCRPC who have progressed despite the use of androgen receptor signaling inhibitors (ARSIs) have limited treatment options. The FDA-approved ^177^Lu-PSMA-617 (Pluvicto; Novartis) is one of the few options available. It has shown an objective response rate of 51% in mCRPC, with a complete response of 9.2% and a partial response of 41.8%, and only extended the median overall survival by 4 months (33). We noted remarkably high ^68^Ga-MY6349 uptake in prostate cancer; consequently, Trop2-targeted ADCs could present a new therapeutic strategy for mCRPC that is resistant to either ARSIs or Pluvicto. Regarding RAIR-DTCs, tyrosine kinase inhibitors (TKIs) are the standard-of-care treatment for patients with RAIR-DTCs; however, treatment options become limited if the disease continues to progress despite TKI therapy. Our findings demonstrated remarkably high and consistent ^68^Ga-MY6349 uptake in most radioactive iodine-refractory PTC lesions. Given that PTC is the most prevalent form of DTC, these results suggest that Trop2-targeting ADCs could be a viable strategy for treating advanced mRAIR-DTCs. These expectations merit further investigation in clinical trials, potentially expanding the treatment landscape beyond the current limitations.

Our clinical PET imaging data demonstrated a marked reduction in radiotracer uptake 24 hours after SG administration in 2 patients. Because of the large molecular weight of ADC, it typically requires at least 24 hours for adequate tumor penetration. As a result, we opted to perform PET/CT imaging with ^68^Ga-MY6349 24 hours after the administration of SG, which would have enabled a more accurate assessment of Trop2 target occupancy by the ADC. Furthermore, it is important to consider that SG, being an ADC, may lead to tumor cell death and thereby reduce the expression of various tumor targets, not only Trop2. This may complicate the interpretation of imaging results, as lower radiotracer uptake could reflect both effective target engagement by SG and potential tumor cell death, resulting in decreased target availability. However, it is noteworthy that even after multiple cycles of SG treatments, the partial response is observed in only a subset of patients. Consequently, we believe that a single administration of SG is unlikely to cause a substantial reduction in target expression, including Trop2, at the 24-hour point, thus minimally impacting the interpretation of our imaging results.

Our study had several limitations. First, the limited number of patients with each type of cancer prevented a subgroup comparison of diagnostic accuracy within the same tumor type. Second, we did not obtain pathological results for Trop2 expression from all participants, thereby limiting our ability to fully investigate the correlation between Trop2 expression and ^68^Ga-MY6349 uptake. Another limitation is that only a small subset of patients with breast cancer underwent Trop2-targeted ADC therapy, which restricted our ability to evaluate the effectiveness of ^68^Ga-MY6349 PET/CT in predicting the clinical outcomes of Trop2-targeted ADC treatment.

In conclusion, the Trop2-targeted radiotracer ^68^Ga-MY6349 has demonstrated its effectiveness to noninvasively assess Trop2 expression in tumors. This facilitates patient screening for Trop2-targeted therapies, including ADC treatment. ^68^Ga-MY6349 PET/CT may offer a new imaging modality for tumor detection and staging, effectively addressing the limitations of ^18^F-FDG PET/CT in certain tumors, particularly thyroid, prostate, and breast cancer. These findings pave the way for more precise cancer diagnosis and treatment.

## Methods

### Sex as a biological variable.

Among the 90 patients enrolled in this study, 38 were male and 52 were female. Female mice were exclusively focused on in this study, and it remains uncertain whether these findings are applicable to male mice. In this study, sex was not considered as a biological variable.

### Preparation of ^68^Ga-MY6349 and its in vitro evaluation.

The Trop2-targeted nanobody MY6349 shares the same core sequence as the nanobody (Nb4) reported by Xu et al. (34), and both nanobodies were produced by Shanghai Novamab Biopharmaceuticals Co. Ltd. However, MY6349 incorporates a GGGC sequence at the C-terminus of Nb4, which was specifically added to facilitate site-specific conjugation with a chelator for radiolabeling purposes. This modification results in the radiolabeling precursor THP-MY6349 (Supplemental Figure 6). In the preparation of ^68^Ga-MY6349, 50 μL (100 μg) THP-MY6349 was added to ^68^GaCl_3_ solution (~4 mL, 740–925 MBq, at pH 5–6) from a germanium-gallium generator. The mixture was then allowed to react for 10 minutes at room temperature, and the radiochemical purity and stability of ^68^Ga-MY6349 were evaluated using radio-HPLC. The sterility tests were performed in-house by the radiochemistry facility of the First Affiliated Hospital of Xiamen University. Overall, ^68^Ga-MY6349 must meet all set criteria before transportation to the clinic for human administration. FITC-MY6349 was also produced for immunofluorescence analysis.

Trop2 expression in 4 cell lines, BxPC-3, HCC1937, C666-1, and A549, was assessed by Western blotting, flow cytometry, and immunofluorescence analysis. The binding specificity of ^68^Ga-MY6349 to Trop2 was determined by cell binding and blocking studies in the 4 cell lines. The binding affinity of ^68^Ga-MY6349 to Trop2 was determined in BxPC-3 cells with high Trop2 expression via a competition binding assay, providing the IC_50_. Furthermore, the dissociation constant value was determined through a saturation binding assay.

The aforementioned procedures are described in detail in Supplemental Methods.

### In vivo evaluation of ^68^Ga-MY6349 in animal models.

Six-week-old BALB/c nude mice acquired from Beijing Vital River Laboratory Animal Technology Co. were housed in a specific pathogen–free facility at the Experimental Animal Center of Xiamen University. Tumor-bearing models were established by subcutaneous injection of 5 × 10^6^ tumor cells (A549, C666-1, HCC1937, or BxPC-3) in 100 μL PBS into the right shoulder of each mouse. PET imaging and biodistribution studies commenced once the diameter of tumors reached 6–10 mm.

For PET imaging, tumor-bearing mice (*n* = 3 per group) were given an intravenous injection of 7.4 MBq ^68^Ga-MY6349. Static PET scans were conducted at 0.5, 1, 2, and 3 hours after injection using an Inveon small-animal PET scanner (Siemens). Blocking studies involved coinjection of 50 nmol unlabeled MY6349 with ^68^Ga-MY6349, followed by scanning at 1 hour after injection. Additional PET imaging was conducted to compare the discrepancies in tumor uptake across 4 different tumor models (*n* = 7 per group). Images were reconstructed using 3D OPMAP 256 (pPetRcn; Siemens Healthineers AG) and analyzed for the percentage of the injected dose per gram of tissue (%ID/g) via region-of-interest delineation.

For biodistribution studies, separate groups of tumor-bearing mice (*n* = 3 per group) were injected with approximately 1.48 MBq ^68^Ga-MY6349, with or without coinjection of the cold MY6349. At predetermined time points after injection, the mice were euthanized, and vital organs and tumors were harvested, weighed, and analyzed for radioactivity.

Mouse tumor tissues were formalin-fixed and paraffin-embedded, then sectioned into 4-μm-thick slices for analysis. The Trop2 IHC staining procedure is outlined in Supplemental Methods.

### Clinical translation of ^68^Ga-MY6349 in patients with various cancer types.

This is a single-center, prospective, investigator-initiated trial to explore the clinical values of ^68^Ga-MY6349 PET/CT. The inclusion criteria were as follows: (a) adult patients (>18 years old) with suspected, newly diagnosed, or previously treated malignancy, (b) no antitumor treatment within 4 weeks prior to the PET/CT scan, and (c) ability to provide informed consent and assent according to the guidelines of the Clinical Research Ethics Committee of the First Affiliated Hospital of Xiamen University. The exclusion criteria included pregnant patients, patients unwilling to provide informed consent, and those with non-malignant disease.

Following previous research on ^68^Ga-based radiopharmaceuticals, each participant received a dose of 3.0–3.7 MBq/kg ^68^Ga-MY6349. One hour after intravenous administration, the participants underwent PET/CT imaging via a hybrid PET/CT scanner (Discovery MI, GE Healthcare). All obtained data were transferred to the Advantage Workstation (version AW 4.7, GE Healthcare) and reconstructed using the Bayesian penalized likelihood reconstruction algorithm (Q.clear, GE Healthcare). The PET/CT scanning and image reconstruction protocols are described in Supplemental Methods. For patients with prostate cancer, additional ^68^Ga-PSMA-11 PET/CT scans were performed as the standard-of-care examination. For patients with other cancer types, additional ^18^F-FDG PET/CT scans were performed for comparative purposes. The PET/CT imaging protocols for ^18^F-FDG and ^68^Ga-PSMA-11 were the same as those for ^68^Ga-MY6349, except that 6 hours of fasting was required before the ^18^F-FDG PET/CT scan (see Supplemental Methods for details).

For the quantitative assessment of radiopharmaceutical uptake, both the SUVmax and the SUVmean were used to evaluate the distribution in normal organs and tumor tissues. The PET/CT scans were evaluated by 2 board-certified nuclear medicine physicians with expertise in interpreting PET/CT data. Uptake was deemed positive if the visually detected area of focal tracer uptake exceeded background levels after physiological uptake, trauma, infection, and inflammatory diseases were excluded (35). Any interpretation discrepancies were resolved by consensus through discussion. The ^68^Ga-MY6349 in vivo distribution pattern was further elucidated through PET imaging at various time intervals across 3 patients to provide a comprehensive overview of the radiotracer behavior in the body. The critical dose for assessing the safety and efficacy of the administered radiopharmaceutical was calculated using OLINDA/EXM software (version 1.1; Hermes Medical Solutions).

### IHC staining of Trop2 expression in tumor specimens from patients with various cancers.

Patient surgical/biopsy specimens were formalin-fixed and paraffin-embedded, then sectioned into 4-μm-thick slices for analysis. The IHC staining of Trop2 is outlined in Supplemental Methods. Trop2 expression on the tumor cell membrane was quantitatively assessed using an IHC staining score, the H score, derived from the formula established in the phase III ASCENT study (10): H score = (3 × % of cells with strong staining) + (2 × % of cells with moderate staining) + (1 × % of cells with weak staining). Trop2 expression was classified into 3 levels based on the H score, with H score ≤ 100, 100 < H score ≤ 200, and H score > 200 indicating low, medium, and high Trop2 expression, respectively.

### Statistics.

Statistical analyses were conducted using SPSS software (version 22.0, IBM). The analyses presented are prospective evaluations of the primary and secondary endpoints. Mean values were compared using a 2-tailed Student’s *t* test. The relationship between the tumor uptake of ^68^Ga-MY6349 (determined by PET quantitative values) and Trop2 expression (determined by IHC staining) was analyzed using simple linear regression. The correlation between the Trop2 expression trichotomy and the ^68^Ga-MY6349 tumor uptake was analyzed using the Kruskal-Wallis test. Comparisons between the SUVmax and TBR obtained from ^68^Ga-MY6349 and other tracer PET/CT scans were performed using Wilcoxon’s paired signed-rank test. Differences in ^68^Ga-MY6349 uptake between the primary tumor and metastatic lesions were analyzed using the Mann-Whitney test. Differences were considered statistically significant at *P* less than 0.05 in a 2-tailed test.

### Study approval.

All animal experiments were approved by the Animal Care and Use Committee of Xiamen University. The clinical study was approved by the Institutional Review Board of The First Affiliated Hospital of Xiamen University and registered at ClinicalTrials.gov under the identifier NCT06188468. Written informed consent was obtained from all participants.

### Data availability.

All data supporting the findings of this study are available within the article and its supplemental information files, including the Supporting Data Values file.

## Author contributions

HC and FW conceptualized the study. LZ, YP, JS, HG, YS, and J Chen performed data curation. LZ, YP, and JS performed formal analysis. HC and FW performed funding acquisition. HF, J Cai, LY, RZ, LS, and HW provided resources. HC, ZW, and FW supervised the study. LZ and YP wrote the original draft. HC and FW reviewed and edited the manuscript.

## Supplementary Material

Supplemental data

ICMJE disclosure forms

Unedited blot and gel images

Supporting data values

## Figures and Tables

**Table 1:**
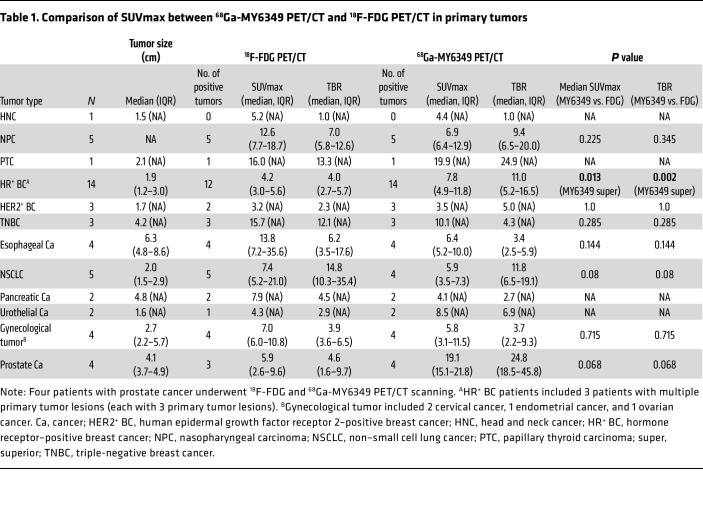
Comparison of SUVmax between ^68^Ga-MY6349 PET/CT and ^18^F-FDG PET/CT in primary tumors

**Table 2:**
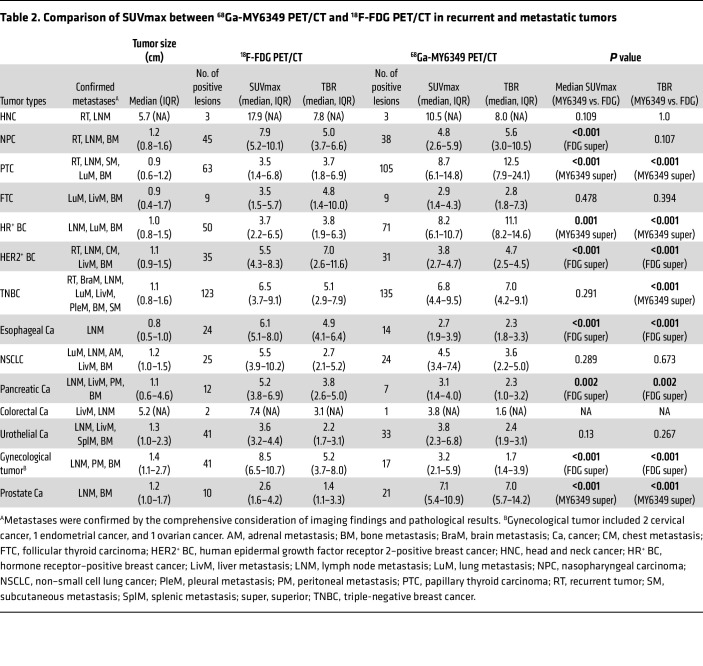
Comparison of SUVmax between ^68^Ga-MY6349 PET/CT and ^18^F-FDG PET/CT in recurrent and metastatic tumors

## Version 1 11/07/2024

In-Press Preview

## Version 2 01/02/2025

Electronic publication
