# Supplementary material for: 68Ga-MY6349 PET/CT imaging to assess Trop2 expression in multiple types of cancer
Source: J Clin Invest. 2024 Nov 7;135(1):e185408. doi: 10.1172/JCI185408 (PMC11684813; doi:10.1172/JCI185408)
Supplement: Supplemental data [file jci-135-185408-s052.pdf]

^{68}Ga-MY6349 PET/CT imaging to assess Trop2 expression in multiple types of cancer

SUPPLEMENTARY MATERIALS

MATERIALS AND METHODS

Materials

Cell lines used for experimentation included A549 (non-small cell lung cancer), C666-1 (EBV-positive nasopharyngeal carcinoma), HCC1937 (TNBC), and BxPC-3 (pancreatic cancer). A549, HCC1937, and BxPC-3 cell lines were sourced from the China National Infrastructure of Cell Line Resources, while C666-1 was obtained from Professor Zhao Chong's research group at the Sun Yat-sen University Cancer Center.

Chemistry

The Trop2-targeting nanobody MY6349 (HuNb1) was prepared according to previously established protocols by Shanghai Novamab Biopharmaceuticals Co., Ltd (reference 35 in the main text). In summary, the Trop2 protein was synthesized and incorporated into a vector, followed by expression in HEK293F cells, purification, and conjugation with adjuvant. Camels were immunized, PBMCs were collected, and mRNA was extracted. A phage display library targeting Trop2 was subsequently generated. Specific nanobodies were identified through screening and validated for their affinity. Selected sequences were cloned, expressed in E. coli, and purified. Moreover, the C-terminus of MY6349 was tagged with GGGC for site-specific Michael addition conjugation. To produce the ^{68}Ga radiolabeling precursor tris

(hydroxypyridinone)-MY6349 (THP-MY6349), 0.35 μmoL of MY6349 was combined with 2 μmoL of THP-Mal (KaiXin Biological, China), 3 μmoL of EDTA-2Na (Sinopharm Chemical Reagent Co., Ltd, China), and 3.5 μmoL of triphenylphosphine-3,3′,3″-trisulfonic acid trisodium salt (TPPTS) (J&K Scientific, China). The pH was adjusted to approximately 8.0, and the reaction proceeded at 37 °C for 4 hours. THP-MY6349 was then purified using an Amicon Ultra-4 Centrifugal Filter (MWCO = 3 kDa, Millipore) with phosphate-buffered saline (pH = 7.0) for 8 cycles. Protein concentration was determined using the BCA method, and the product was confirmed by MALDI-TOF mass spectrometry (ultrafleXtreme, Bruker, USA). The synthesized THP-MY6349 was subjected to mass spectrometry (MALDI-TOF) for validation, revealing a molecular weight of approximately 13.73 kDa for the native nanobody and approximately 14.67 kDa for THP-MY6349 post-conjugation with Mal-THP (Supplemental Figure 1).

Take an appropriate amount of the nanobody MY6349 and start by passing the sample through a 10 kDa ultrafiltration centrifuge tube. Centrifuge at 4000 g and 4 degrees Celsius, concentrating the sample to 250 μL. To ensure thorough purification, add 2 mL of 1x PBS, centrifuge again, and reduce the volume back to 250 μL. Repeat this process twice more. After the final concentration step, you should have about 300 μL of concentrated solution. To measure the antibody concentration, take 5 μL from this solution, dilute it by a factor of 50, and use the BCA assay. For conjugation with FITC, dissolve MY6349 and FITC-NHS (R-FC-005, Stargraydye, China) in 400 μL of PBS at a 1:15 molar ratio. Adjust the pH to 8.0 using 0.1 M sodium carbonate-sodium bicarbonate buffer (pH 9.2). Allow the mixture to react at room temperature for 2 hours. Once the reaction is complete, purify the sample using a PD10

column, eluting with 1x PBS. Add an additional 4 mL of 1x PBS to ensure complete elution. Collect the purified solution in 1.5 mL centrifuge tubes, gathering 6 drops per tube. Finally, measure the antibody concentration using the BCA assay. The final concentration of FITC-MY6349 in this experiment is 2.6 mg/mL.

Radiochemistry

After labeling, the ^{68}Ga-MY6349 solution was filtered through a 0.22 μm sterile filter membrane, collected in sterile vials, and subjected to endotoxin and sterility tests following the methods described in the 2020 Edition of the *Chinese Pharmacopoeia*.

The radiochemical purity (RCP) of ^{68}Ga-MY6349 was assessed using a high-performance liquid chromatography system (Agilent 1260 Infinity II) equipped with a Superdex 200 Increase 10/300 GL column (Cytiva) and a radioactive detector (Raytest Socket81030111). Phosphate-buffered saline (pH = 7.4) served as the mobile phase, flowing at a rate of 0.4 mL/min.

In vitro stability of ^{68}Ga-THP-MY6349 injection was evaluated in phosphate-buffered saline (pH = 7.4) and fetal bovine serum (FBS). Specifically, 0.5 MBq (50 μL) of ^{68}Ga-MY6349 was mixed with either 200 μL of 5% FBS or 200 μL of PBS buffer (0.01 M, pH 7.4) and incubated at room temperature for 4 hours. The mixtures were analyzed by radio-HPLC to assess *in vitro* stability.

***In vitro* cell assays**

For *in vitro* cell uptake studies, tumor cells were cultured in 24-well plates until reaching

approximately 80% confluence. Following this, the regular medium was replaced with FBS-free medium for subsequent experiments. The cellular uptake assay involved treating four cell lines (A549, C666-1, HCC1937, and BxPC-3) with ^{68}Ga-MY6349 alone or in combination with 10 nmol of a blocking agent (unlabeled MY6349) or full-length Trop2 antibody (BioXCell, Cat. No. BE0408) for 60 minutes to assess the specificity and efficacy of ^{68}Ga-MY6349 uptake. To determine the binding affinity of ^{68}Ga-MY6349 to Trop2, the half-maximal inhibitory concentration (IC_{50}) and the equilibrium dissociation constant (K_D) were assayed. For the IC_{50} assay, after reaching 80% confluence, BxPC-3 cells were treated with various concentrations of unlabeled MY6349-THP (1.3×10^{-5} to 10^{-13} M; n=3) using ^{68}Ga-MY6349 as the radioligand. Following a 60-minute incubation, the cells were washed with cold PBS supplemented with 2% FBS, harvested, and the radioactivity was measured using a gamma counter. Furthermore, a saturation binding assay was conducted using 96-well multiscreen filter plates (Millipore, Ireland). A total of 200,000 cells were incubated in 200 μL of phosphate-buffered saline (PBS) containing 2% fetal bovine serum (FBS) with a range of ^{68}Ga- MY6349 concentrations (1~50 nM) for 2 hours under ice-cold conditions. Nonspecific binding was assessed in the presence of 6 μg of cold MY6349. Following incubation, the cells were washed with cold PBS supplemented with 2% FBS, harvested, and the radioactivity was measured using a gamma counter. The IC_{50} and K_D values were analyzed using Prism v7.0 (GraphPad Software Inc., San Diego, Canada). Each experiment was performed with three replicates for each condition.

Western blotting, flow cytometry, immunofluorescence, immunohistochemical staining of Trop2

For western blotting assay, proteins were extracted with lysis buffer (150 mM NaCl, 50 mM Tris-HCl [pH 8.0], 1mM EDTA and 1% of the protease inhibitor and the phosphatase inhibitor). Approximately 20 μg of total protein per sample was separated by SDS-PAGE and transferred to a PVDF membrane (Millipore). The membranes were pre-incubated with 5% skimmed milk in TBST for 1 h, followed by incubation with human Trop2 antibody (CST, Cat. No. 47866). Membranes were washed with TBST three times and incubated with horseradish peroxidase-labelled secondary antibody (Abclonal, Cat. No. AS039), which was detected using an enhanced chemiluminescence detection system (CLINX, ChemiScope 6200).

Regarding the flow cytometry, viable cells were counted and resuspended in cell staining buffer at a concentration of 5–10 × 10^6 cells/mL. Next, 100 μL/tube of cell suspension (5–10 x 10^5 cells/tube) was distributed into 2-mL plastic tubes. Fc receptors were then blocked by pre-incubation in 2.5 μg of purified anti-human CD16/CD32 mAb (BD PharmingenTM, Cat. No. 564219) per 10^6 cells in 100 μL at room temperature for 10 min. Then, 100 μL of cells per tube were incubated with fluorescent full-length mAbs specific to human Trop2 (BioLegend, Cat. No. 363804) or fluorescent nanobody (FITC-MY6349-THP) for 30 min at 2 –8 °C. The tubes were then washed once in 2 mL of stain buffer and centrifuged at 350 × g for 5 min. The cells were analyzed and data acquired using the BD FACSCanto II Flow Cytometer.

For immunofluorescence staining, tumor cells were seeded in confocal dishes. The samples were then fixed with 4% paraformaldehyde for 10 minutes. Subsequently, the cells were washed thrice with PBS and blocked with 10% goat serum for 30 minutes to minimize

nonspecific binding. The cells were then incubated overnight with the primary antibody, full-length mAbs anti–Trop2 (CST, Cat. No. 47866) or fluorescent nanobody (FITC-MY6349), at a dilution of 1/200, followed by three washes with PBS. Afterward, the cells with full-length mAbs were stained with the secondary antibody, AF594–conjugated IgG (Abclonal, Cat. No. AS039), for one hour and washed three times with PBS. Nuclei were stained blue using DAPI (UE, Cat. No. D4080). To evaluate the specificity and binding efficacy of FITC-MY6349 to the Trop2 protein on the cell surface, we used a standard full-length Trop2 antibodies as controls. Specifically, the anti-Trop2 antibody #363804 (BioLegend, USA) was selected for flow cytometry and the anti-Trop2 antibody #47866 antibody (CST, USA) served as the primary antibody for immunofluorescence experiments.

For histological analysis, tissue specimens underwent a series of preparations. They were fixed in 10% buffered formalin, dehydrated through a series of ethanol washes, embedded in paraffin, and subsequently stained using Immunohistochemistry (IHC). The paraffin-embedded samples were dewaxed in ethanol and subjected to antigen retrieval using 0.01 mol/L sodium citrate containing 0.05% Tween. IHC was performed on paraffin-embedded mouse tissues using primary antibody targeting Trop2 antibody (abcam, Cat. No. ab214488) an'd second antibody (Yuanxibio, Cat. No.H-D110041-100T), in accordance with our previously established protocol (reference 36 in the manuscript). Pictures were taken with the Aperio Versa 8 tissue imaging system (3D HISTECH).

Establishment of tumor bearing mice

All animal experiments were approved by the Animal Care and Use Committee of Xiamen University. Six-week-old BALB/c nude mice acquired from Beijing Vital River Laboratory Animal Technology Co. (Beijing, China) were housed in a Specific Pathogen Free (SPF) facility at the Experimental Animal Center of Xiamen University. Tumor-bearing models were developed by subcutaneously injecting 5×10^6 tumor cells (A549, C666-1, HCC1937, or BxPC-3) in 100 μL PBS into the right shoulder of each mouse. PET imaging and biodistribution studies commenced once tumors reached 6-10 mm^3.

The PET/CT scanning and image reconstruction protocols

The dose of intravenously injected ^{18}F-FDG, ^{68}Ga-MY6349, ^{68}Ga-PSMA-11 was calculated according to the patient's weight (3.7 MBq [0.1 mCi]/kg) for FDG; 1.8-2.2 MBq [0.05-0.06 mCi]/kg for ^{68}Ga-MY6349, ^{68}Ga-PSMA-11). Data were acquired using a hybrid PET/CT scanner (Discovery MI, GE Healthcare, Milwaukee, WI, USA) after 1 h of intravenous administration. All scans were performed according to the protocol as we have previously described (reference 36 in the manuscript). Briefly, a PET scan was immediately performed after the CT scan in 3-dimensional acquisition mode with 6-8 bed positions and 2.0-2.5 min/position. All the obtained data were transferred to the Advantage Workstation (version AW 4.7, GE Healthcare, Milwaukee, WI, USA); data were reconstructed using the Bayesian penalized likelihood (BPL) reconstruction algorithm (Q.clear, GE Healthcare, Milwaukee, WI, USA), with a penalisation factor (beta) of 500. The reconstructed images were then co-registered and displayed.

Supplemental Figures

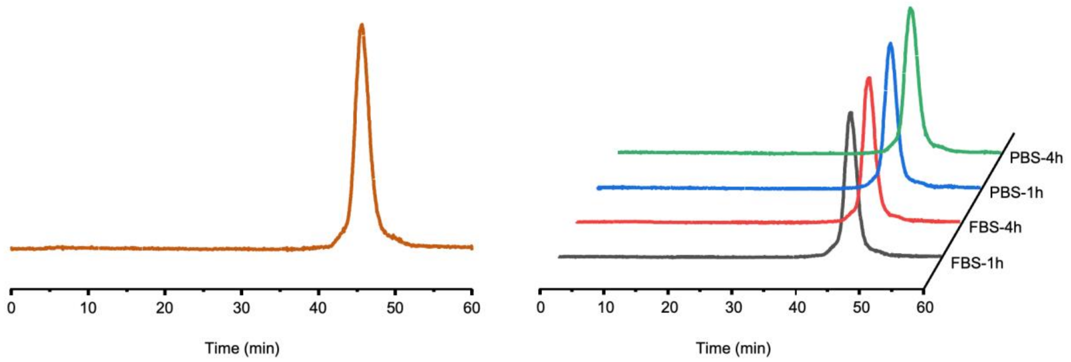

Supplementary Figure S1. *In vitro* stability analysis of ^{68}Ga-MY6349 in phosphate buffer saline (PBS) and fetal bovine serum (FBS).

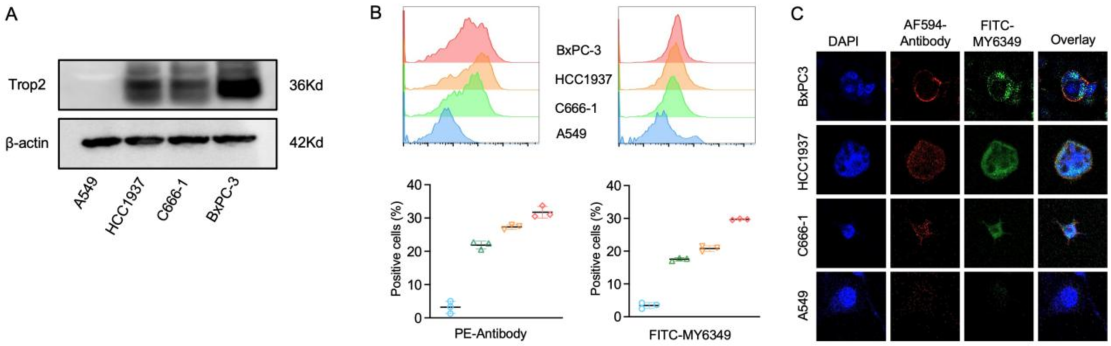

Supplementary Figure S2. Evaluating Trop2 expression by standard full-length Trop2 antibody and MY6349 nanobody. (A) Trop2 expression on A549, C666-1, HCC1937 and BxPC-3 cells, as determined by western blotting. (B) Trop2 expression on A549, C666-1, HCC1937 and BxPC-3 cells, as determined using flow cytometry. (C) Confocal images of immunofluorescence staining using a standard full-length Trop2 antibody and FITC-labeled MY6349.

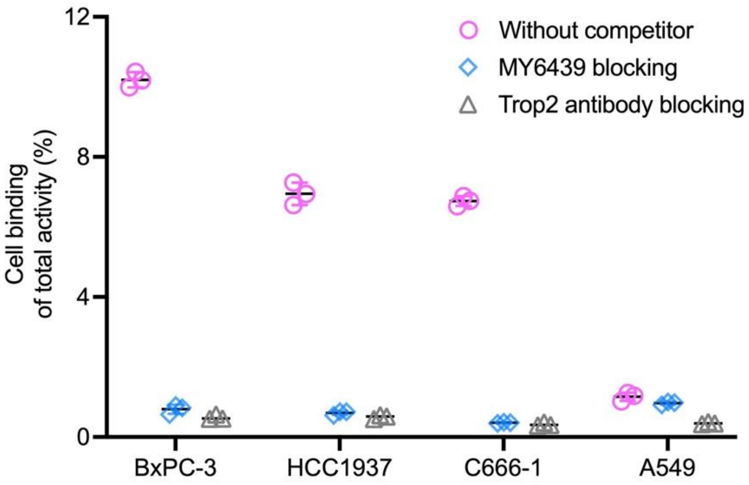

Supplementary Figure S3. Cell binding assay of ^{68}Ga-MY6349 to A549, C666-1, HCC1937 and BxPC-3 cells, alongside a blocking experiment using an unlabeled MY6349 or full-length Trop2 antibody to validate the binding specificity (n = 3/group).

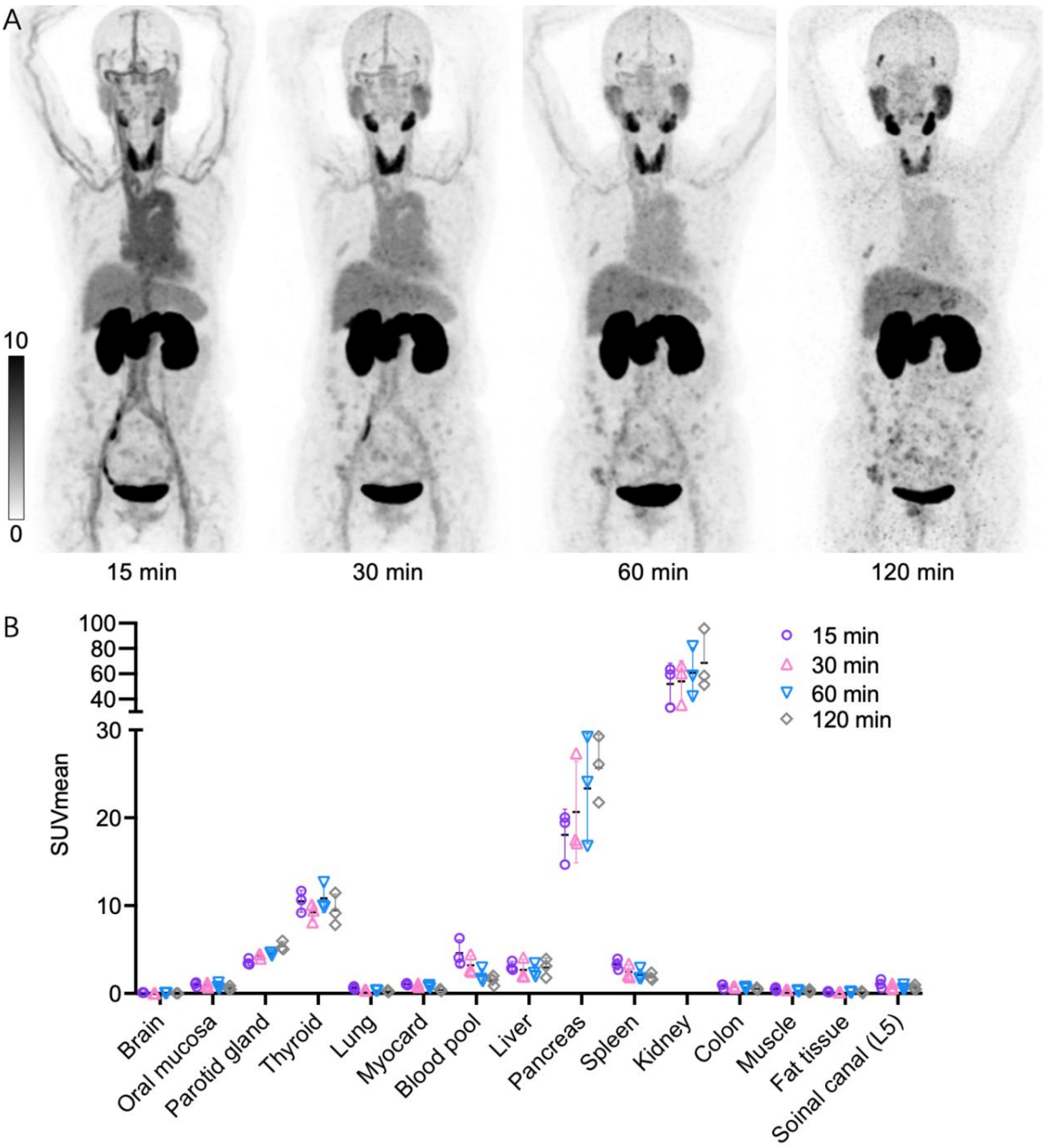

Supplementary Figure S4. Representative PET imaging of ^{68}Ga-MY6349 (A) and the corresponding SUVmean values of normal organs (B) at 15-,30-, 60-, and 120-min post-injection in three patients. The patients with pancreatic cancer image from Figure 4 is shown again in Supplementary Figure S4.

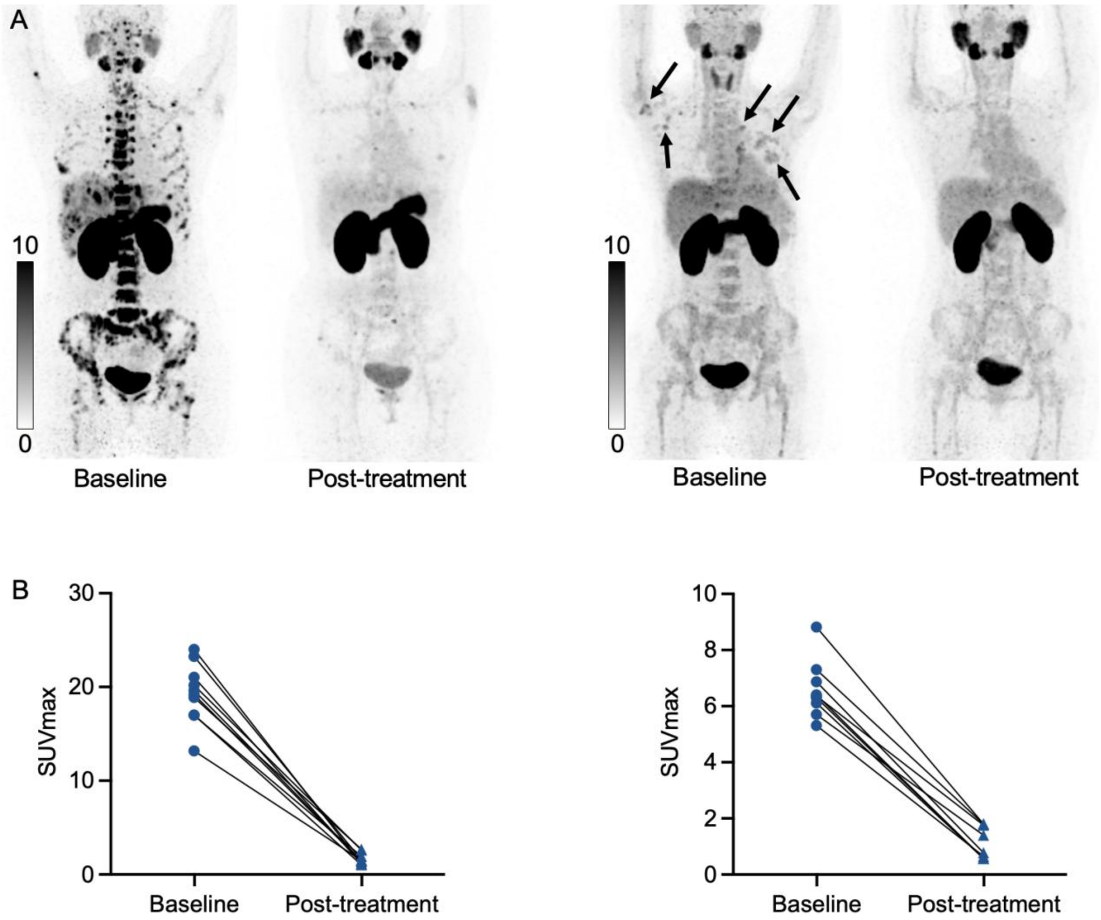

Supplementary Figure S5. (A) Representative maximum-intensity projection images obtained using ^{68}Ga-MY6349 PET/CT before and 24 h after SG treatment. (B) Lesion-by-lesion comparison in the same patient, using paired ^{68}Ga-MY6349 PET/CT images taken before and 24 h after SG treatment.

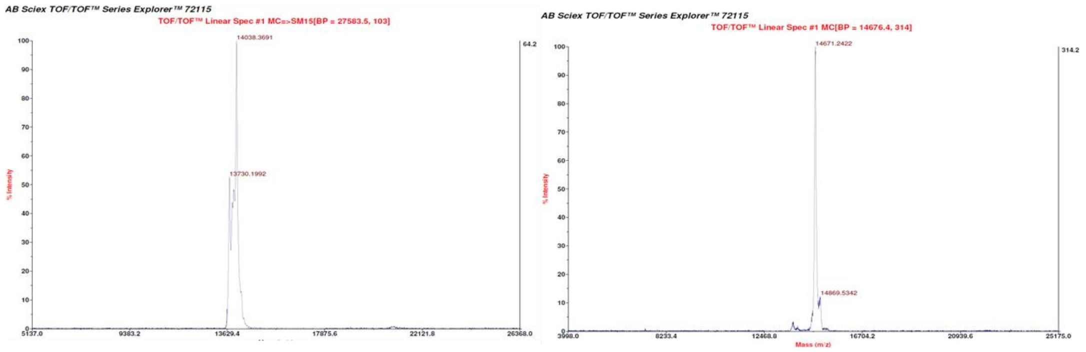

Supplementary Figure S6. The synthesized MY6349 was subjected to mass spectrometry for validation, revealing a molecular weight of approximately 13.73 kDa for the native nanobody and approximately 14.67 kDa for MY6349 post-conjugation with Mal-THP.

Supplementary Tables

Supplementary Table S1. Participant characteristics

Characteristics	Number
**Number of participants**	90
**Age (y)**	
Median (IQR)	59.0 (49.0–67.0)
**Sex**	
Male	40
Female	50
**Types of cancer***	
Head and neck cancer (HNC)	3
Nasopharyngeal carcinoma (NPC)	7
Papillary thyroid carcinoma (PTC)	13
Follicular thyroid carcinoma (FTC)	3
Medullary thyroid carcinoma (MTC)	3
HR+ breast cancer (HR+ BC)	11
HER2+ breast cancer (HER2+ BC)	5
Triple-negative breast cancer (TNBC)	12
Esophageal cancer	4
Non-small cell lung cancer (NSCLC)	7
Pancreatic cancer	2
Colorectal cancer	2
Gynecologic tumor†	4
Prostate cancer	16
Urothelial cancer	3
**Clinical questions for PET/CT imaging**	
Initial staging of cancer	45
Recurrence detection	45
**Final diagnosis**	
Histopathological confirmation (via biopsy or surgery)	67
Diagnostic imaging/follow-up	23

* Five patients were diagnosed with synchronous double cancers: one with nasopharyngeal carcinoma and prostate cancer, two with non-small cell lung cancer and prostate cancer, one with HR+ breast cancer (three primary lesions of the same pathological type) and cervical cancer, and one with HER2+ breast cancer and non-small cell lung cancer. Additionally, two breast cancer patients had three primary breast cancer lesions each, all of the same pathological type, which were classified as one cancer types in the statistical analysis. Initial tumor staging included one patient with occult breast cancer.

† Gynecologic tumor included two cervical cancers, one endometrial cancer, and one ovarian cancer.

Four patients with prostate cancer underwent ^{18}F-FDG and ^{68}Ga-MY6349 PET/CT scanning. 12 patients with prostate cancer underwent ^{68}Ga-PSMA-11 and ^{68}Ga-MY6349 PET/CT scanning. IQR = interquartile range.

Data are numbers, unless indicated otherwise.

Supplementary Table S2. Summary of effective doses for ^{68}Ga-MY6349 using OLINDA/EXM.

**Target organ**	**Mean** (mSv/MBq)	**SD** (mSv/MBq)
Adrenal glands	3.72E–04	8.96E–05
Brain	8.51E–07	2.93E–07
Breasts	3.04E–05	6.27E–06
Gallbladder Wall	-	-
LLI Wall	4.24E–04	5.34E–05
Small Intestine	1.73E–05	5.14E–06
Stomach Wall	5.03E–04	8.93E–05
ULI Wall	1.06E–05	1.17E–06
Heart Wall	-	-
Kidneys	8.11E–04	1.81E–04
Liver	1.14E–03	4.83E–04
Lungs	4.28E–04	8.75E–05
Muscle	9.62E–06	2.93E–06
Ovaries	2.57E–04	2.23E–04
Pancreas	3.17E–03	8.43E–04
Red Marrow	3.10E–04	6.56E–05
Osteogenic Cells	1.96E–05	1.92E–05
Skin	6.82E–06	6.31E–07
Spleen	4.87E–05	1.36E–05
Thymus	-	-
Thyroid	2.12E–03	1.88E–03
Urinary Bladder Wall	2.20E–03	8.16E–04
Uterus	3.47E–04	5.91E–04
Effective Dose Equivalent	3.81E–02	5.91E–03
Effective Dose	1.46E–02	2.32E–03

LLI, lower large intestine; SD, standard deviation; ULI, upper large intestine.

Supplementary Table S3. Summary of ^{18}F-FDG and ^{68}Ga-MY6349 uptake in patients with differentiated thyroid cancers

Patient No.	Patient Status	Pathological type	^{18}F-FDG PET-derived SUVmax (IQR)	^{68}Ga-MY6349 PET-derived SUVmax (IQR)
Patient No. 1	Radioiodine-refractory	PTC	2.7-5.3	6.8-16.1
Patient No. 2	Radioiodine-refractory	PTC	7.1-13.0	5.9-13.0
Patient No. 3	Radioiodine-refractory	PTC	1.7-8.3	5.1-12.2
Patient No. 4	Radioiodine-refractory	PTC	0.6-1.4	8.6-16.8
Patient No. 5	Radioiodine-refractory	FTC	1.9-4.9	3.3-5.8
Patient No. 6	Radioiodine-refractory	PTC	3.6-16.4	5.5-15.2
Patient No. 7	Radioiodine-refractory	PTC	3.2-12.7	6.2-17.9
Patient No. 8	Radioiodine-refractory	PTC	1.3-1.5	5.7-9.2
Patient No. 9	Radioiodine-refractory	PTC	1.2-12.5	3.5-26.3
Patient No. 10	Radioiodine-refractory	PTC	2.9-6.9	5.8-33.0
Patient No. 11	Radioiodine-refractory	PTC	1.6-5.8	6.5-33.9
Patient No. 12	Radioiodine-refractory	PTC	1.2-1.3	7.6-10.6
Patient No. 13	Radioiodine-refractory	FTC	1.0-4.0	2.9-4.3
Patient No. 14	Radioiodine-refractory	FTC	2.5-14.7	1.2-1.8
Patient No. 15	Radioiodine-refractory	PTC	1.7-2.3	6.7-37.4
Patient No. 16	Initial staging	PTC	5.7-13.6	4.1-16.1

PTC, papillary thyroid cancer; FTC, follicular thyroid cancer; IQR = interquartile range.

Supplementary Table S4. Comparison of SUVmax on ^{68}Ga-MY6349 and ^{68}Ga-PSMA-11 PET/CT in patients with prostate cancer

Patients with Prostate Ca	n	Tumor Size (cm)	^{68}Ga-PSMA-11 PET/CT			^{68}Ga-MY6349 PET/CT			P value	
		Median (IQR)	No. of positive lesions	SUVmax (Median, IQR)	TBR (Median, IQR)	No. of positive lesions	SUVmax (Median, IQR)	TBR (Median, IQR)	Median SUVmax (MY6349 vs. PSMA-11)	TBR (MY6349 vs. PSMA-11)
Primary tumor	7	3.7 (2.5-4.1)	7	23.3 (12.3-49.4)	19.7 (12.3-44.2)	7	24.5 (13.1-37.2)	22.3 (10.9-33.8)	0.612	0.735
Recurrent tumor	5	2.3 (1.7-3.4)	5	4.9 (3.5-16.3)	5.0 (3.2-17.6)	5	6.1 (4.3-24.3)	12.2 (8.7-34.6)	**0.043** (MY6349 Super)	**0.043** (MY6349 Super)
Metastases (LNM, BM)	204	1.4 (1.0-2.3)	161	11.5 (4.8-18.5)	11.9 (3.8-20.9)	183	12.3 (7.8-17.1)	15.7 (9.1-25.3)	0.222	**<0.001** (MY6349 Super)

12 patients with prostate cancer underwent ^{68}Ga-PSMA-11 and ^{68}Ga-MY6349 PET/CT scanning. LNM = lymph node metastasis, BM = bone metastasis. IQR = interquartile range.
