# Supplementary figures and images for: 68Ga-MY6349 PET/CT imaging to assess Trop2 expression in multiple types of cancer
Source: J Clin Invest. 2024 Nov 7;135(1):e185408. doi: 10.1172/JCI185408 (PMC11684813; doi:10.1172/JCI185408)

Full unedited gel for Figure S2A

Trop2 36 Kd

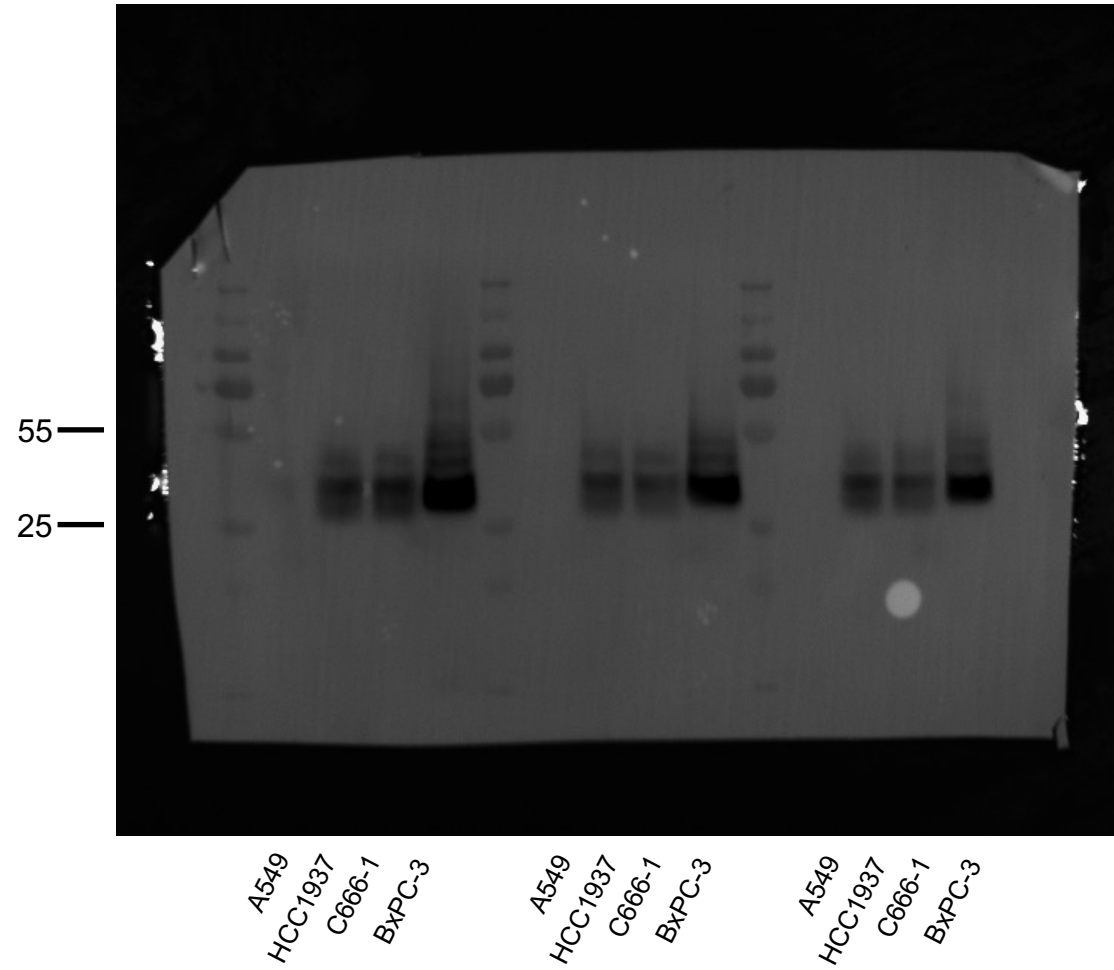

$\beta$ -actin 42 Kd

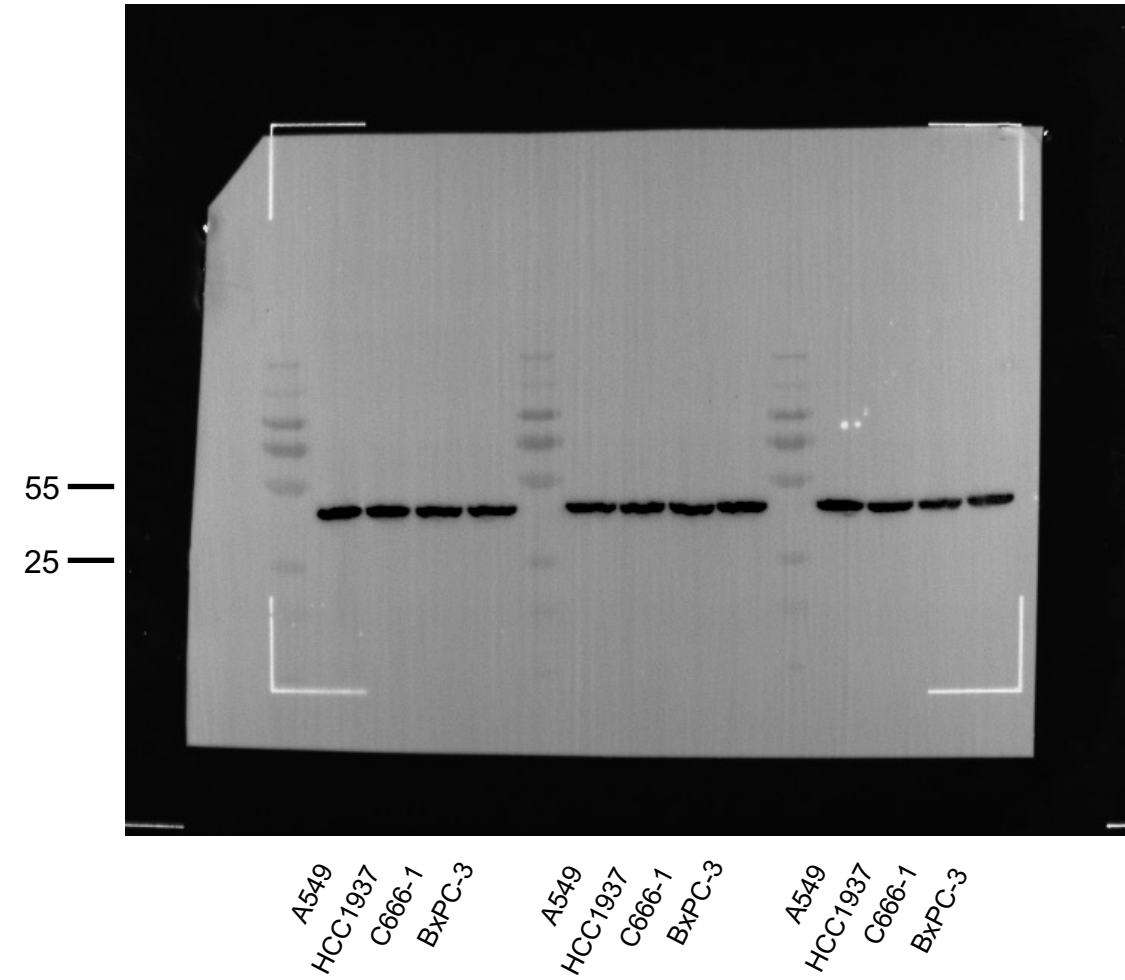

Supplement: Unedited blot and gel images [file jci-135-185408-s054.pdf]
